# RamEx: an R package for high-throughput microbial ramanome analyses with accurate quality assessment

**DOI:** 10.1186/s40168-026-02339-3

**Published:** 2026-02-10

**Authors:** Yanmei Zhang, Gongchao Jing, Rongze Chen, Yanhai Gong, Yuandong Li, Yongshun Wang, Xixian Wang, Jia Zhang, Yuli Mao, Yuehui He, Xiaoshan Zheng, Mingchao Wang, Hao Yuan, Jian Xu, Luyang Sun

**Affiliations:** 1 Single-Cell Center, CAS Key Laboratory of Biofuels, Shandong Key Laboratory of Energy Genetics, Qingdao New Energy Shandong Laboratory, Shandong Energy Institute, Qingdao Institute of Bioenergy and Bioprocess Technology, Chinese Academy of Sciences, Qingdao, 266101 China; 2 Laboratory for Marine Biology and Biotechnology, Qingdao Marine Science and Technology Center, Qingdao, 266104 China; 3 College of Life Sciences, University of Chinese Academy of Sciences, Beijing, 100049 China; 4 Qingdao Single-Cell Biotechnology Co., Ltd, Shandong, Qingdao 266101 China

**Keywords:** Ramanome, Microbial phenotype, Quality assessment, High-throughput profiling, Analysis pipeline, Microbial community

## Abstract

**Background:**

Microbial single-cell Raman spectroscopy (SCRS) has emerged as a powerful tool for label-free phenotyping, enabling rapid characterization of microbial diversity, metabolic states, and functional interactions within complex communities. However, high-throughput SCRS datasets often contain spectral anomalies from noise and fluorescence interference, which obscure microbial signatures and hinder accurate classification. Robust algorithms for outlier detection and microbial ramanome analysis remain underdeveloped.

**Results:**

Here, we introduce RamEx, an R package specifically designed for high-throughput microbial ramanome analyses with robust quality control and phenotypic classification. At the core of RamEx is the Iterative Convolutional Outlier Detection (ICOD) algorithm, which dynamically detects spectral anomalies without requiring predefined thresholds. Benchmarking on both simulated and real microbial datasets—including pathogenic bacteria, probiotic strains, and yeast fermentation populations—demonstrated that ICOD achieves an F1 score of 0.97 on simulated datasets and 0.74 on real datasets, outperforming existing approaches by at least 19.8%. Beyond anomaly detection, RamEx provides a modular and scalable workflow for microbial phenotype differentiation, taxonomic marker identification, metabolic-associated fingerprinting, and intra-population heterogeneity analysis. It integrates Raman-based species-specific biomarkers, enabling precise classification of microbial communities and facilitating functional trait mapping at the single-cell level. To support large-scale studies, RamEx incorporates C++ acceleration, GPU parallelization, and optimized memory management, enabling the rapid processing of over one million microbial spectra within an hour.

**Conclusions:**

By bridging the gap between high-throughput Raman-based microbial phenotyping and computational analysis, RamEx provides a comprehensive toolkit for exploring microbial ecology, metabolic interactions, and antibiotic susceptibility at the single-cell resolution. RamEx is freely available under the MIT license at https://github.com/qibebt-bioinfo/RamEx.

Video Abstract

**Supplementary Information:**

The online version contains supplementary material available at 10.1186/s40168-026-02339-3.

## Background

Single-cell Raman spectroscopy (SCRS) has emerged as a powerful tool for microbial metabolic phenotyping, as enabling label-free, non-invasive characterization of microbial cells based on their unique vibrational “fingerprints” [1–3]. By capturing metabolic states at the single-cell level, SCRS has become an essential component of the expanding microbial single-cell “-omics” landscape [4, 5], enabling direct investigation of complex microbial communities, ranging from microbial identification [6–8] and biomass quantification [9–11] to environmental stress evaluation [12, 13] and metabolic heterogeneity analysis [14, 15]. The collection of single-cell Raman spectra obtained from a microbial population or consortium under defined conditions is termed a “Ramanome”, which represents a biochemical phenotype landscape of individual cells within that system, thereby serving as a single-cell-resolved spectral dataset that enables quantitative assessment of phenotypic heterogeneity and metabolic interactions. With single-cell research generating unprecedented volumes of data, recent technological advances in high sensitivity detectors [16, 17] and microfluid-based cell sorting Raman measurement systems [18–22] have significantly enhanced the throughput and scalability of SCRS. Notably, modern Raman platforms now enable high-throughput microbial phenotyping, capturing thousands of Raman spectra per minute [23, 24], enabling the rapid acquisition of large-scale microbial Raman datasets [25–27]. Concurrently, the development of standardized Raman spectral databases [28] has facilitated comparative microbial analyses and reproducibility across studies**.** These studies highlight the potential of Raman-activated cell sorting for identifying and screening key players in targeted processes [29, 30]. However, despite these technological strides, computational tools to extract meaningful insights from the deluge of microbial Raman data have remained underdeveloped, presenting critical bottlenecks to the field.

Several software packages and algorithms have been developed to support microbial Raman spectral data analysis, including both commercial platforms and open-source tools (Supplementary Table 1). Commercial platforms such as LabSpec, WiRE, and WITec are user-friendly and allow non-specialists to perform basic preprocessing and spectral feature identification. However, they are largely confined to elementary preprocessing tasks and lack the scalability and advanced data mining capabilities required for large-scale, high-dimensional biological applications [31, 32]. Open-source packages, such as MCR-ALS [33], MALDIquant [34], and RamanSpy [35], provide greater flexibility and support more complex workflows, particularly in leveraging community-driven resources and facilitating seamless integration with modern framework. In addition, deep-neuron network has been used to perform the preprocessing steps for Raman spectrum and observed considerable results with traditional methods [36–38]. However, existing tools are not specifically optimized for microbial ramanome analysis, particularly in addressing key challenges such as microbial population clustering, phenotypic heterogeneity, and metabolic interaction networks [39]. An effective approach for handling large-scale ramanome data is to adapt methodologies from other omics disciplines, particularly single-cell sequencing. For instance, scRNA-seq analysis tools such as Seurat [40], BayesSpace [41], and Monocle [42] have been applied for tissue structure reconstruction [43], and chemometric spectral unmixing techniques have been repurposed for compositional analysis [33]. However, these adapted approaches often fail to capture the unique characteristics of microbial Raman spectra, such as continuous spectral signals with baseline variations, distinctive noise patterns, and overlapping signals [44, 45]. Unlike single-cell transcriptomics, which operates on sparse, zero-inflated sequencing data, microbial ramanome data follows a fundamentally different statistical distribution, making direct adaptation of sequencing-based approaches suboptimal for microbial spectral analysis [46, 47].

Furthermore, due to the inherent detection characteristics of Raman spectroscopy, various spurious signals such as thermal noise, shot noise, black-body radiation from samples, fluorescence from cellular structural features, cell motion artifacts, and environmental fluctuations can easily distort the weak signals originating from biological tissues [48–50]. Although the optimized design of optical system elements can reduce the frequency of these irrelevant signals to some extent, such interference remains unavoidable during automated high-throughput data acquisition [51, 52]. Improper removal of these anomalous spectra can severely compromise downstream phenotype analyses, particularly in complex microbial communities [39, 53, 54]. However, there are few outlier detection algorithms specifically designed for large-scale microbial ramanomes. Most existing methods [55–57] rely on empirically determined thresholds that require manual optimization for different experimental conditions, making them poorly suited for the complex, heterogeneous nature of microbial spectral data [58]. While machine learning approaches [50, 59], particularly deep learning methods, have demonstrated promising results in spectral analysis but necessitate extensive training datasets and careful hyperparameter optimization [6, 60], limiting their generalizability across different microbial communities, experimental platforms, and environmental conditions.

To address these pressing challenges in high-throughput microbial Raman spectral analysis, we present Ramanome Explorer (RamEx), an integrated computational framework specifically designed for large-scale microbial ramanomes processing and analysis. At its core, RamEx introduces novel algorithmic advancements for robust spectral quality control, microbial phenotype differentiation, and metabolic-associated fingerprinting, optimizing data extraction from high-dimensional microbial Raman spectra. Its scalable architecture ensures efficient analysis of large and diverse microbial datasets, as demonstrated in benchmarking tests across eight biological datasets (cumulative > 1 million spectra) spanning multiple microbial taxa and environmental conditions (Supplementary Table 2). RamEx provides a standardized yet flexible analytical framework, combining automated data processing pipelines with customizable workflow modules to accommodate a broad range of microbial applications.

By bridging the current technical and infrastructural gaps in microbial Raman-omics, RamEx represents a crucial step towards establishing Raman flow cytometry as a mainstream technology, and opens new possibilities for large-scale functional microbial profiling at unprecedented scales. Importantly, RamEx was conceived not only as a computational platform but as a biological discovery tool, enabling microbiologists to explore the mechanisms that shape microbial population structure and metabolic dynamics. To demonstrate its biological relevance, we evaluated RamEx in two representative application scenarios: (i) single-cell phenotypic heterogeneity within isogenic populations, and (ii) population-level differentiation and inter-species metabolic characteristics.

## Results

### RamEx provides a versatile multifaceted framework for microbial ramanome analysis

Understanding complex and high-dimensional single-cell Raman spectroscopy datasets presents significant challenges due to issues like high collinearity, nonlinearity, and the presence of outliers. To address the challenges in analyzing and interpreting ramanome data, we developed RamEx, an R package designed specifically to analyze large-scale Raman datasets using novel algorithms and comprehensive single-cell analysis workflows. It features: (*i*) an outlier detection algorithm that operates without prior knowledge or fixed criteria; (*ii*) optimized clustering and marker identification algorithms adapted to the unique properties of high dimensional Raman spectra; (*iii*) curated computational framework with tools and pipelines for key Raman tasks such as cell type/species identification, clustering phenotypic analysis, antibiotic resistance detection and molecular composition analysis; (*iv*) enhanced computing efficiency through C++ optimization and GPU computing; and (*v*) standardized Raman dataset format with integrated metadata and evaluation metrics. RamEx is freely available at GitHub (https://github.com/qibebt-bioinfo/RamEx).

**Fig. 1:**
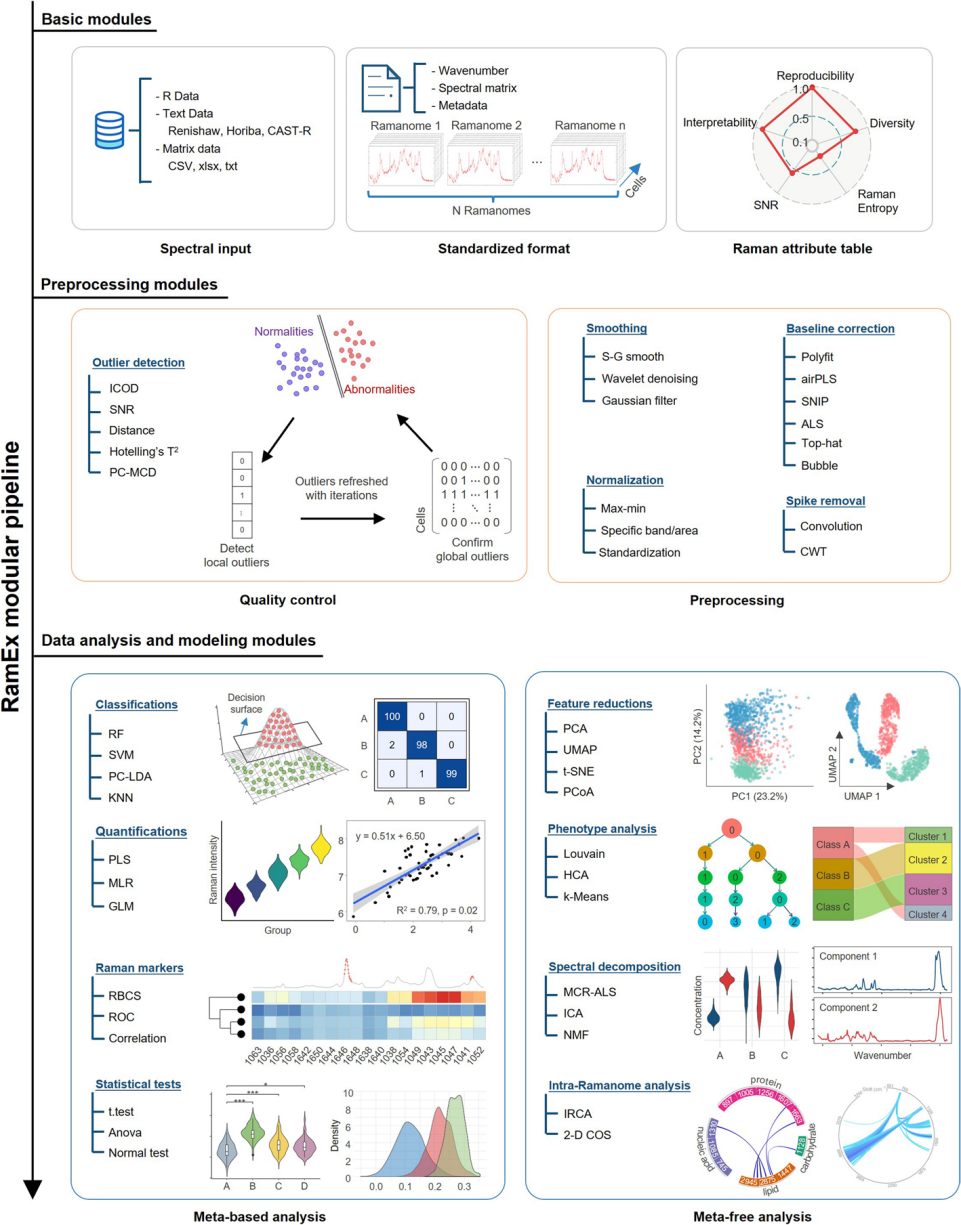
Schematic overview of the RamEx computational framework. The framework comprises three core modules: basic module (input/output management and data format standardization and evaluation), preprocessing modules (quality control and preprocessing steps), and data analysis and modeling modules (classification, quantification, marker identification, phenotype analysis, spectral decomposition and intra-ramanome analysis)

RamEx is structured into three core modules: the basic module, the spectral preprocessing module, and the data analysis and modeling module (Fig. 1), encompassing a variety of useful analytical functionalities in metabolic phenotyping of cells (Supplementary Table 3). First of all, the basic module handles data import, data evaluation, and data management, supporting cross-platform input formats from mainstream instrument manufacturers such as Horiba, Renishaw, Thermo Fisher Scientific, WITec, and Bruker. It efficiently manages both single-point data collection, where each spectrum is stored in a separate file, and mapping data enriched with coordinated information. The module automates data type organization, eliminating the need for user intervention or configuration. It also introduces the Raman Attribute Table, which assesses datasets based on reproducibility, interpretability, Raman entropy, signal-to-noise ratio (SNR), and diversity. This tool enables users to efficiently evaluate the quality and potential of large datasets for subsequent analysis (see Methods; Supplementary Fig. 1).

Moreover, the preprocessing module includes a novel outlier detection algorithm we developed called Iterative Convolutional Outlier Detection (ICOD), which enhances data quality by effectively identifying aberrant spectra without requiring empirical threshold settings. RamEx also provides methods for detecting other common outliers, offering flexibility and adaptability in quality control (QC). Beyond outlier detection, the module offers a carefully selected range of classic spectral preprocessing tools, including spike removal, smoothing, baseline correction, and normalization. Multiple methods are provided for each step to suit specific dataset characteristics [61–64]. Users can customize methods and parameters for comprehensive adjustment or utilize a standardized workflow for rapid analysis, ensuring adaptability and efficiency in processing Raman spectra.

Finally, the data analysis and modeling module provides a suite of functions supporting the primary applications of ramanomics, broadly categorized into meta-based (supervised) and meta-free (unsupervised) applications (Fig. 1). For meta-based analyses, RamEx offers classification methods such as Principal Component Analysis–Linear Discriminant Analysis (PCA-LDA), Random Forests (RF), Support Vector Machines (SVM), and K-Nearest Neighbors (KNN). These classifiers are particularly useful for species/strains identification, physiological state discrimination, and the recognition of environmental or stress responses based on distinctive Raman signatures. Quantitative analysis tools like Partial Least Squares Regression (PLS), Multiple Linear Regression (MLR), and Generalized Linear Regression (GLR), can be applied to estimate intracellular levels of specific metabolites or macromolecules from spectral features. Marker identification is optimized for both qualitative and quantitative scenarios, enabling users to locate Raman bands correlated with specific phenotypes (e.g., lipid accumulation, stress adaptation, or antibiotic resistance). This can be achieved via both machine learning-based recognitions (e.g., Raman Barcode of Cellular-response to Stresses, RBCS [65]) or univariate identifications based on band traversal (e.g., correlation-based or ROC-based approaches).

For meta-free applications, the module includes dimensionality reduction algorithms such as Uniform Manifold Approximation and Projection (UMAP) and t-distributed Stochastic Neighbor Embedding (t-SNE), which are exceptional for visualizing high-dimensional, highly correlated, and subtly varying biological spectra. Unsupervised clustering algorithms provided include Gaussian Mixture Model (GMM), hierarchical clustering, Louvain, and Leiden algorithms are available to identify latent phenotypic subgroups within microbial populations without prior annotation, helping to uncover functionally distinct subcommunities. Unique to spectral analysis, RamEx incorporates spectral decomposition methods such as Multivariate Curve Resolution–Alternating Least Squares (MCR-ALS), Independent Component Analysis (ICA), and Non-negative Matrix Factorization (NMF) for chemical interpretations, which group spectral components according to co-varying intensities to infer pure biochemical substances or functional modules. Additionally, for intra-ramanome analysis, RamEx offers both Intra-Ramanome Correlation Network Analysis (IRCA) algorithm and Two-Dimensional Correlation Spectroscopy (2D-COS), specifically designed to capture dynamic metabolic interconversions by analyzing correlation patterns among spectral intensities, thereby revealing metabolic interaction processes and biochemical dependencies within single-cell populations [66].

It is worth noting that supervised modules (e.g., classification and quantification) require explicit validation using separate test datasets, whereas non‑model‑based modules (e.g., quality control, dimensionality reduction, phenotype analysis, and marker identification) are evaluated through biological reproducibility across independent replicates rather than a conventional train–test split. Overall, RamEx offers a comprehensive suite of user-friendly tools, strategically integrated to facilitate the effective analysis and interpretation of complex Raman data. The subsequent section demonstrates the practical application of these methods.

### Robust quality control in RamEx ensures high-fidelity microbial ramanome analysis

**Fig. 2:**
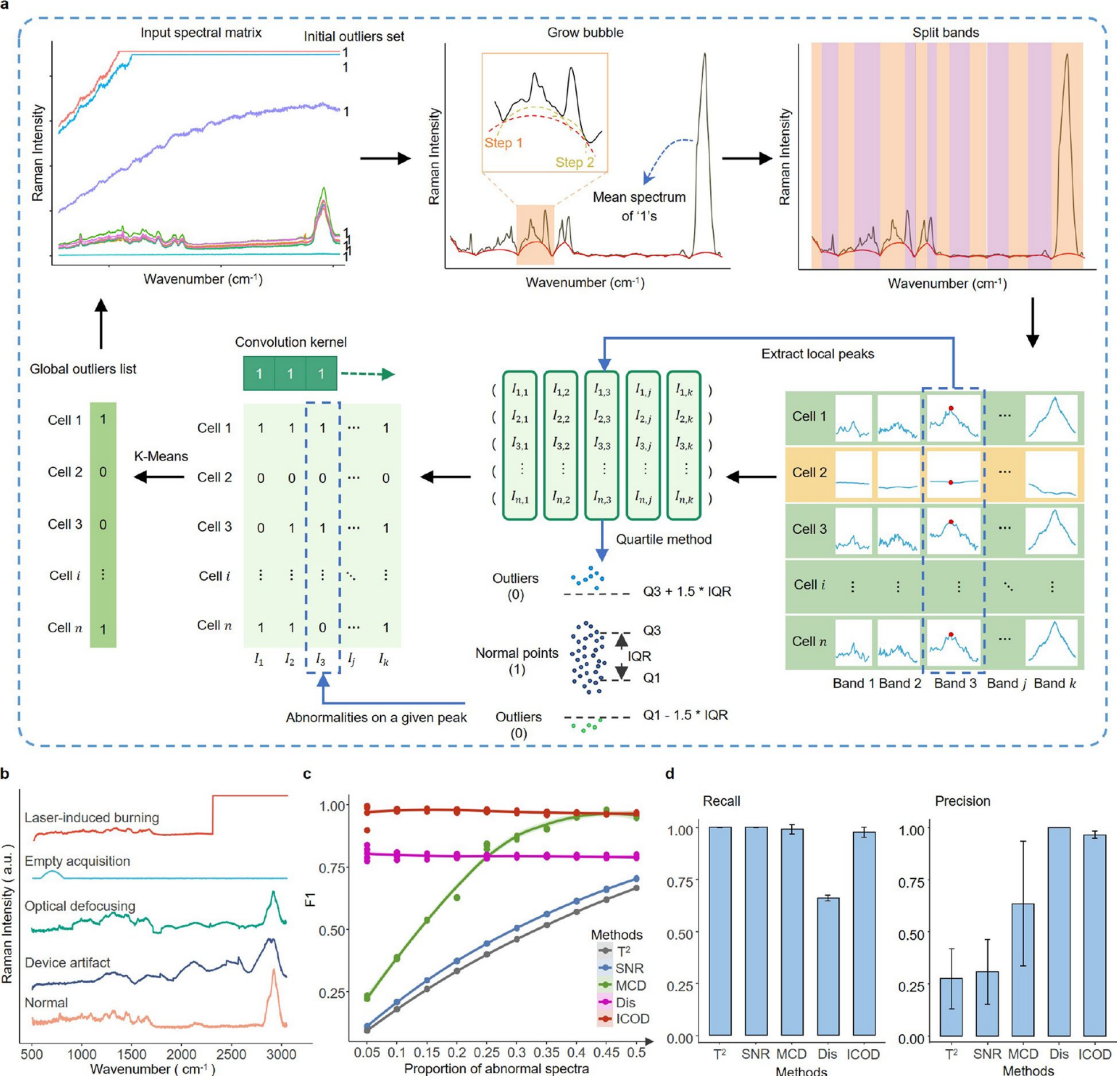
ICOD is robust to various quality levels. **a**, Schematic illustration of ICOD highlighting key computational steps. **b**, Types of simulated anomaly spectra. **c**, F1 scores of five outlier detection methods across datasets with varying proportions of abnormalities. **d**, Recall and precision of different outlier detection methods, where dots represent means; error bars indicate standard deviations, *n* = 5 independent datasets across 10 quality levels

Identifying abnormal spectral acquisitions is crucial for enhancing data quality in biological single-cell datasets. Currently, this process often involves using a combination of filtering criteria and adjusting empirical thresholds tailored to specific projects. The challenge of setting these thresholds intensifies when integrating datasets from multiple batches, as batch variations require meticulous calibration to ensure consistent and reliable analysis outcomes. In single-cell Raman spectroscopy, biological Raman spectra generally display consistent dimensions (number of bands) and similar patterns across different groups, resulting in a lack of obvious variation. This similarity complicates automated quality assessment, such as spectral similarities and quality parameters, which rely on pre-defined thresholds. Moreover, these criteria are highly empirical and often require expert judgment. To address this, we developed the ICOD algorithm in RamEx, which dynamically identify and remove low-quality Raman spectra iteratively, without requiring context-specific empirical criteria. By assuming that most biological spectra within a population share consistent overall features, it iteratively identifies Raman outliers in an unsupervised manner, with unbiased consideration of all meaningful peaks (Fig. 2a; Methods).

Specifically, the ICOD method employs an iterative unsupervised clustering approach to effectively identify and classify outliers in single-cell Raman spectra. Initially, peak identification is conducted using a modified bubble method [67], which extracts intensity information at peak positions, and reduces the dataset from thousands of features to a few dozen, thus preserving critical spectral information while mitigating background noise. The original dataset ${X}_{n\times m}$ will be transformed into ${X}^{\prime}_{n\times p}$ ($p\ll m$). Subsequently, the quartile outlier method is applied to detect anomalies at each peak, where an observation ${x}_{ij}$ is considered a local outlier if ${x}_{ij}<{Q1}_{j}-1.5\times {IQR}_{j}$ or ${x}_{ij}>{Q3}_{j}+1.5\times {IQR}_{j}$. This process converts the dataset into a Boolean matrix ${B}_{n\times p}$, where ${B}_{ij}=1$ if ${x}_{ij}$ is an outlier, and ${B}_{ij}=0$ otherwise. Then k-means clustering ($k=2$) and convolution filtering are subsequently applied to distinguish normal and anomalous spectra, with convolution mitigating the impact of isolated local anomalies and enhancing the clustering robustness. To prevent progressive over-rejection of samples, spectra flagged as outliers are excluded from recalculating peak and quantile statistics at each iteration, but remain included in the clustering process. The global outlier list is iteratively updated until it stabilizes between cycles. Optionally, if the proportion of anomalous peaks among the remaining spectra exceeds an internally defined threshold, additional refinement rounds are performed. This iterative refinement progressively enhances the distinction between normal and anomalous spectra by accounting for the inherent redundancy in Raman signals.

To demonstrate the performance of ICOD, we conducted a comparative analysis against several established outlier detection techniques, including threshold-based approaches like Euclidean distance [55] and signal-to-noise ratio (SNR) [56] (to remove samples with low similarity and low SNR), statistics-based approaches like Hotelling’s T^2^ [59, 68] and Principal components—minimum covariance determinant (PC-MCD) [69] (to remove samples with low confidence), using both simulated large-scale datasets and real benchmarking studies (Methods). Four kinds of common anomalous spectra [55]—laser-induced burning, empty acquisition, optical defocusing, and device artifact—along with high-quality Raman spectra, were generated using a Gaussian process regression model (Fig. 2b, Supplementary Fig. 2a). Each test dataset consisted of 10,000 simulated Raman spectra, with the proportion of anomalous spectra varying from 5 to 95%, resulting in 19 distinct datasets. These anomalous spectra were randomly selected from the four afore-mentioned categories. To ensure reproducibility, five replicates were created for each specific percentage of anomalous spectra, culminating in a total of 95 datasets.

Notably, ICOD outperformed other outlier detection methods, consistently achieving a superior F1 score of 0.97 (Fig. 2c). In contrast, other methods were sensitive to the proportion of outliers, performing poorly at low outlier ratios. Specifically, Hotelling’s T^2^, SNR, and PC-MCD exhibited overly stringent criteria, misclassifying many spectra, while the Euclidean distance method was too permissive. ICOD demonstrated high accuracy and precision attributed to its iterative convergence process, detecting over 75% of outliers by the second epoch and the rest in subsequent iterations (Supplementary Fig. 2b), reflecting ICOD’s adaptive learning rate and robust convergence. Gradient testing revealed ICOD’s enhanced performance at outlier compositions below 70%, applicable to most scenarios (Fig. 2c and Supplementary Fig. 2c). This robust performance depends on the assumption that normal spectra constitute the largest and most coherent group in the dataset. At each iteration, ICOD excludes outliers from statistical recalculations but retains them for clustering, allowing the majority to define the reference. However, if abnormal spectra exceed a certain proportion (empirically around 70% in our tests), they begin to dominate the clustering process, leading to misclassification of the true normal group as outliers. This limitation occurs only under extreme conditions and is unlikely in most practical datasets. In situations where ambiguous spectra predominate, preliminary QC is recommended to ensure normal data remains the majority, thereby maximizing ICOD’s effectiveness.

**Fig. 3:**
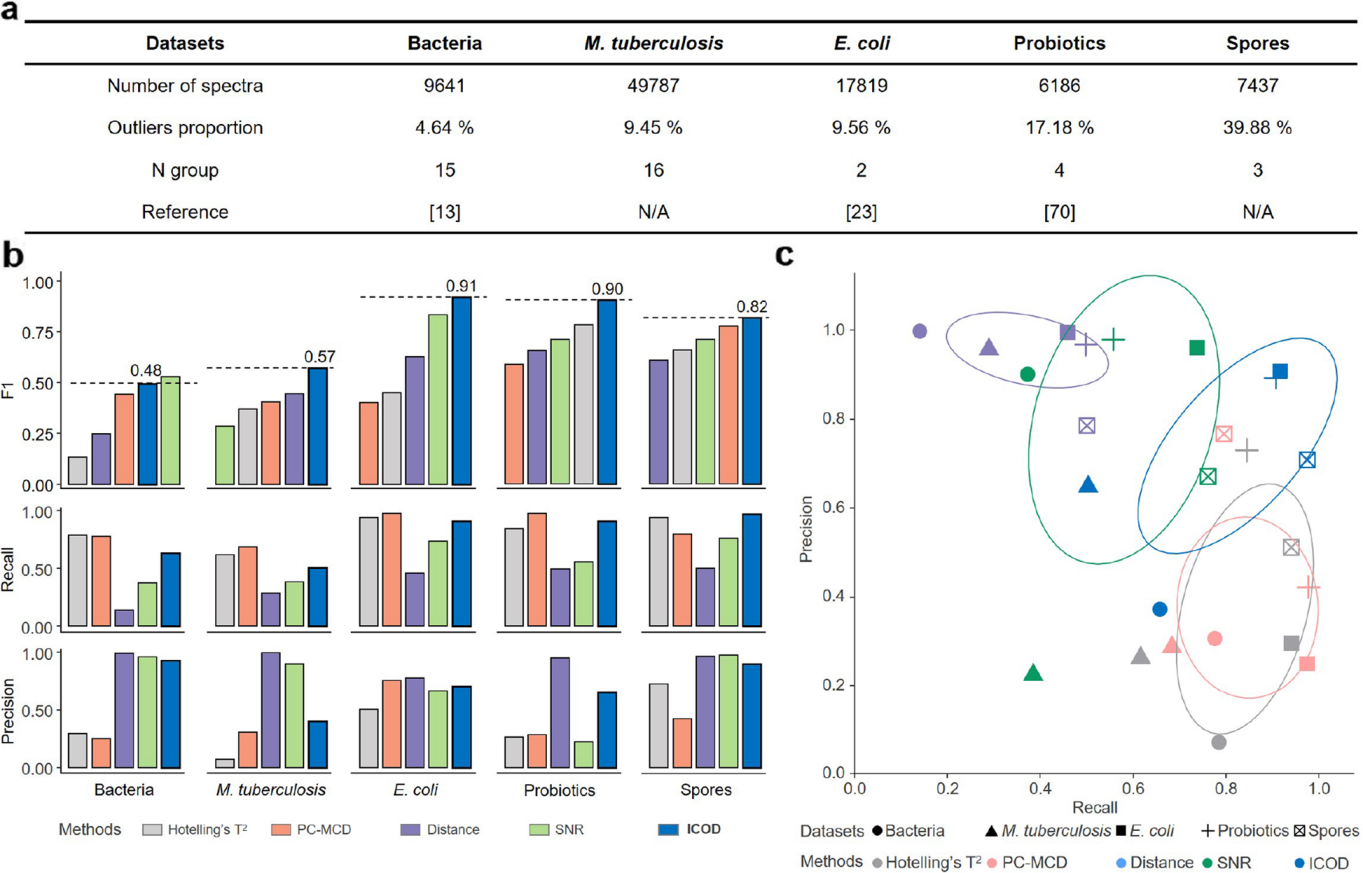
ICOD demonstrates superior performance over other methods on five public datasets. **a**, Summary of the five public datasets (for detailed descriptions, see Methods). **b**, F1 scores, recalls and precisions of five outlier detection methods on real datasets, the ground truth served as an artificial, item-by-item determination. **c**, Scatter plot showing the precision and recall value for the five datasets, where ICOD is always at a higher level than that obtained by other methods, demonstrating its superior performance and robustness

To further benchmark ICOD’s performance under real-world conditions, we analyzed three publicly available ramanome datasets, along with two self-collected datasets (Fig. 3a). The datasets involved data collected from various Raman instruments, species, and outlier proportions, featuring dry smear samples comprising 9,641 spectra from pathogenic bacteria [13], 49,787 spectra from *Mycobacterium abscessus* under drug exposure, and 6,186 spectra from multiple probiotic strains [70]. Additionally, these datasets included 17,819 spectra from *Escherichia coli* under various treatments [23] and 7,437 spectra related to chlorflavonin production, both obtained using Raman flow cytometry. When compared to expert consensus-determined outlier labels, ICOD consistently outperformed competing algorithms across these datasets in terms of F1 scores, achieving an average score of 0.74, 19.8% higher than the next best method (mean F1 score: 0.61 of SNR method) (Fig. 3b). Further analysis shows that ICOD effectively balanced precision and recall in outlier detection across these five datasets (Fig. 3c), whereas Hotelling’s T^2^ and PC-MCD were overly stringent, resulting in lower data retention rates, and methods based on distance and SNR lacked sensitivity to anomalies (Fig. 3b). These results demonstrated the reliability and adaptability of ICOD as a leading method among those tested. While peak-based anomaly detection can be affected by baseline removing, tests using three widely used baseline-correction methods demonstrated that ICOD maintained superior performance than other alternative methods (Supplementary Fig. 3). Notably, although the SNR method achieved a better F1 score on the “Bacteria” dataset, a small number of outliers still existed, distorting the overall PCA score space as shown in Supplementary Fig. 4. In contrast, the datasets processed by ICOD exhibited clearer inter-group differences and tighter intra-group clustering based on the results of reduction and Adonis analysis. We next tested the generalizability of ICOD beyond microbial data by extending our validation to mammalian cells. A public dataset with 4,772 spectra from human neuronal cells were used [71]. The algorithm maintained robust performance, achieving a high F1 score of 0.93 compared to expert manual annotation (Supplementary Fig. 5a). This result confirms ICOD’s core assumption that, most valid biological spectra share a foundational Raman spectrum profile, also applies to mammalian cells.

We further challenged ICOD in a highly heterogeneous context using a self-collected marine environmental microbiome dataset with 5,131 spectra, where it still achieved a strong F1 score of 0.84 (Supplementary Fig. 5b). However, this analysis demonstrated a typical trade-off of unsupervised algorithms. While ICOD effectively removing technical artifacts, it also flagged a rare group of carotenoid-rich microalgae as outliers because their unique spectral signatures deviated significantly from the statistically dominant microbial population (Supplementary Fig. 5c, d). To address such cases, a heterogeneity-aware quality-control framework was recommended (Methods; Supplementary Fig. 6a). The framework first computes a Heterogeneity Index (HI) to assess whether a dataset behaves a single dominant Raman mode or contains multiple subpopulations. HI is computed by combining cluster-size entropy with inter-cluster separation, where low values indicate homogeneity and high values indicate intrinsic heterogeneity. For datasets with high HI, RamEx performs coarse clustering and applies ICOD within each subpopulation, preventing minority biological groups from being removed simply due to their deviation from the dominant mode. Finally, RamEx visualizes rejected spectra to enable optional expert-guided data rescue. This framework was validated using a synthetic heterogeneous dataset constructed by merging the marine microbiome dataset, probiotics dataset (with taxonomically diverse in previous tests), and an extra carotenoid-rich microalgae dataset. This mixture exhibited high intrinsic heterogeneity (HI = 0.91). Applying stratified ICOD substantially improved quality-control performance: the initial F1 score increased from 0.61 (global ICOD) to 0.79, and further to 0.82 after expert-guided data rescue (Supplementary Fig. 6b, c). A similar improvement was observed when applying global ICOD followed by expert rescue, confirming that expert-guided refinement is important for highly heterogeneous datasets.

In summary, ICOD demonstrated robust performance across both simulated and real-world datasets. However, F1 scores alone cannot fully reflect the impact of quality control on subsequent data analysis. To further illustrate ICOD’s benefits, we next applied it to new datasets and evaluated its impact on downstream analyses within RamEx.

### RamEx supports high-resolution single-cell biochemical profiling of microbial communities

**Fig. 4:**
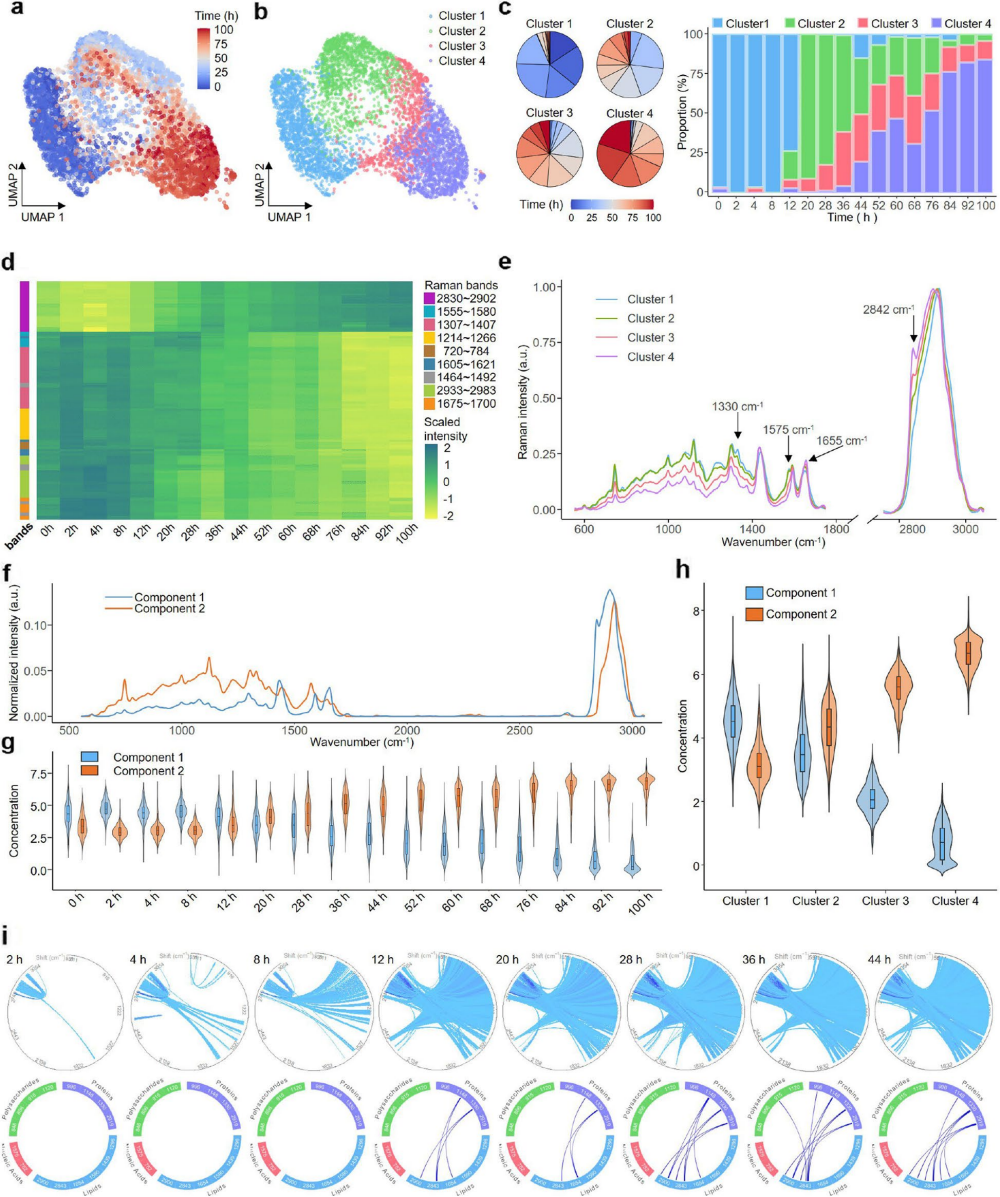
Application of RamEx to ramanomes from yeasts reveals intracellular substance changes over fermentation time. **a** and **b**, Uniform Manifold Approximation and Projection (UMAP) reduction of all ramanomes, colored by fermentation time or unsupervised clustering results. **c**, Time point composition of each cluster and cluster composition at each time point. Cluster 1 only appears in the first 12 h, while cluster 4 emerges after 36 h and increases over time. **d**, Heatmap of single markers shows the Raman intensity trends over time, identified from single-cell spectra. **e**, Mean spectra of 4 clusters, recorded by Raman markers derived from population-averaged spectra. **f** and** g**, Pure component spectral profiles and the corresponding concentration profiles resolved by MCR-ALS, though their concentrations show a more significant trend with cluster variation in (**h**). **i**, Global-IRCN and local-IRCN at each time point, with connections between Raman bands colored by their correlations

To demonstrate RamEx’s capacity for biological interpretation, we analyzed spectral datasets spanning the fermentation process of *Saccharomyces cerevisiae*. By integrating nonlinear dimensionality reduction with unsupervised clustering, RamEx effectively resolved the subtle variations inherent in ramanome data, optimized by parallel computing to handle large-scale datasets. UMAP results revealed distinct structures associated with culture time (Fig. 4a), underscoring the effectiveness of RamEx in capturing dynamic biological changes. Furthermore, Louvain clustering partitioned the population into four distinct phenotypic states based on their ramanome-derived phenotypes (Fig. 4b). The temporal progression of these clusters aligns with fermentation stages, demonstrating RamEx’s ability to dissect single-cell heterogeneity and map the metabolic evolution of the population. Subsequent composition analysis explicitly quantified the spatiotemporal dynamics of each cluster (Fig. 4c).

Extracting biologically interpretable markers is critical for decoding cellular states. RamEx provides two complementary strategies for this purpose: direct correlation and coherence-removed baseline correction. The direct correlation approach captured continuous metabolic transitions, revealing distinct kinetic patterns including gradual increases, decreases, and sharp drops (Fig. 4d). While this method captures global intracellular shifts, it also reflects substantial cell-to-cell heterogeneity, resulting in overlap between time points that obscures fermentation stages (Supplementary Fig. 7b, c). To mitigate this, RamEx computes markers as intensity pairs, which effectively reduces such overlap by prioritizing relative biochemical changes over absolute intensities (Supplementary Fig. 7 d, e).

Complementarily, the coherence-removed method addresses baseline shifts caused by variations in adjacent peaks, efficiently eliminating spectral regions lacking chemical relevance. This method accurately identifies emerging (e.g., 1655 cm⁻^1^, 2842 cm⁻^1^) and diminishing (e.g., 1330 cm⁻^1^, 1575 cm⁻^1^) peaks during fermentation (Fig. 4e, Supplementary Fig. 8). These markers correspond to well-characterized biochemical features, including unsaturated fatty acids (1655 cm⁻^1^), C–H stretching in TAGs (2842 cm⁻^1^), C–H deformation in proteins (1330 cm⁻^1^), and nucleic acids (1575 cm⁻^1^) (Supplementary Table 4) [20, 44, 72]. Collectively, this analysis elucidates the biological rationale behind cell clustering, confirming a metabolic trajectory characterized by the accumulation of fatty acids and TAGs concomitant with a decrease in protein and nucleic acid levels.

In addition to individual markers, RamEx employs MCR-ALS to deconvolute complex intracellular spectra. Unlike single-marker analysis, this unsupervised approach treats each spectrum as a linear combination of components, enabling the separation of lipid concentrations from other cellular substances (Fig. 4f). Despite cell-to-cell variability, this decomposition reveals nuanced concentration trends across fermentation timeframes (Fig. 4g) and accentuates distinct compositional variations between clusters (Fig. 4h), offering significant insights into the evolution of subpopulations within the fermentation system.

To capture the interplay of phenotypic traits, RamEx incorporates the Intra-Ramanome Correlation Network (IRCN) [66]. Leveraging inherent metabolic heterogeneity, IRCN maps intricate relationships within a single population snapshot. We observed that global IRCN density increased with fermentation time, indicating more active intracellular metabolite transformations (Fig. 4i, top). Additionally, the local-IRCN focuses on changes at specific peaks rather than across all spectral bands, providing a more nuanced understanding of specific metabolite conversion (Fig. 4i, bottom). By capturing these dynamics without requiring traditional experimental gradients, RamEx highlights the value of analyzing individual developmental trajectories.

Lately, we emphasized the critical importance of quality control by re-evaluating all downstream analyses using alternative QC methods in place of ICOD. The results demonstrated that RamEx significantly enhanced the clarity of phenotypic patterns by reducing data variance and improving the separation between different time points (Supplementary Fig. 9, 10). ICOD proved to be particularly effective in maintaining the integrity of biological conclusions, offering more coherent dimensionality reduction, improved clustering structure, and more reliable Raman markers compared to the SNR-based approach (Supplementary Fig. 11, 12). Furthermore, the anticipated increase in IRCN activity with fermentation time was consistently tracked, reflecting predictable potential metabolite conversions (Supplementary Fig. 13). Spectral decomposition was notably improved, with MCR-ALS achieving successful convergence, thereby enhancing effectiveness of the decomposition process. These outcomes underscore the robustness of the quality control methods.

### RamEx enables high-throughput and scalable microbial ramanome analyses

**Fig. 5:**
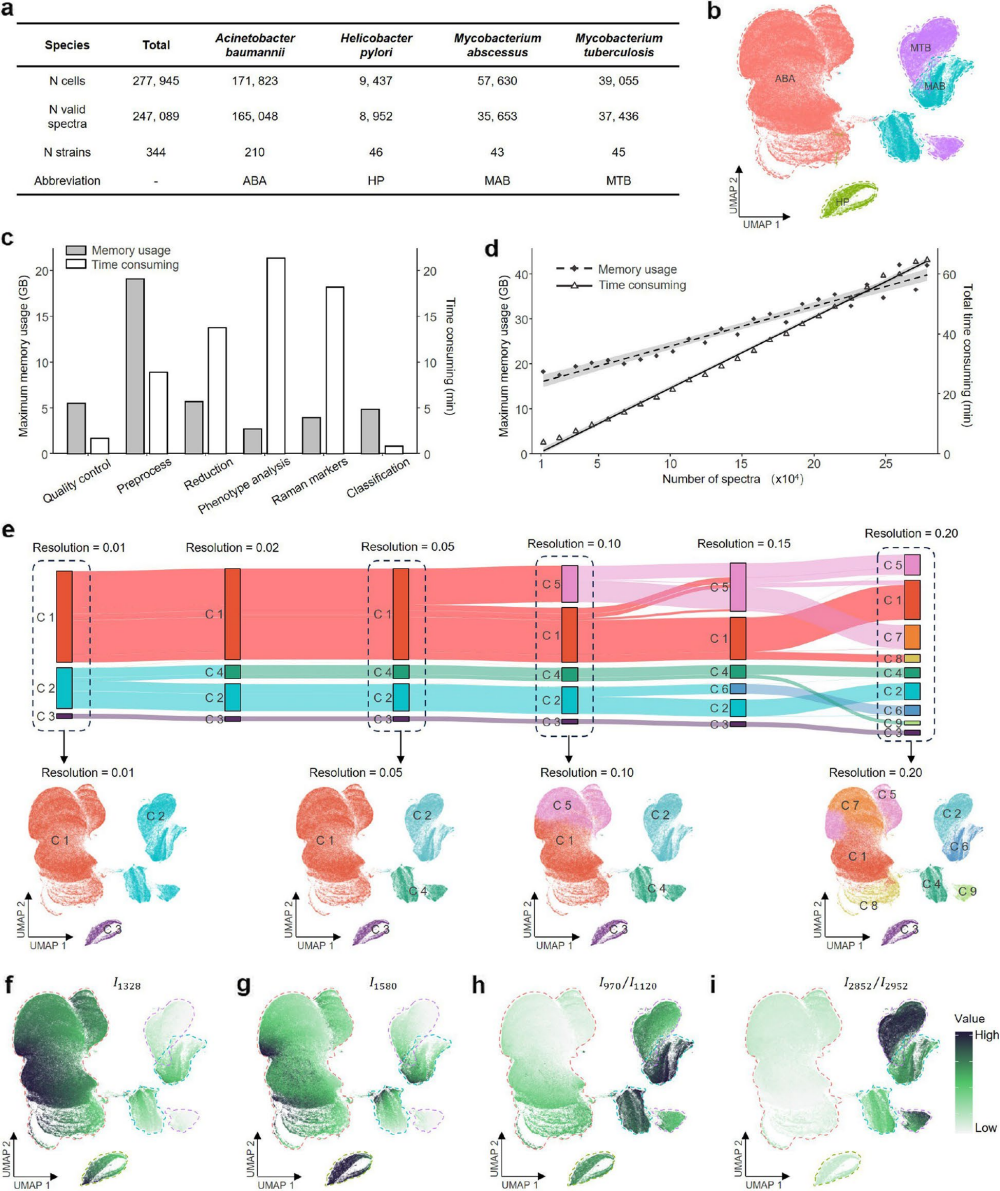
Application of RamEx to a large-scale ramanome dataset demonstrates high throughput and ability to unravel subtle yet important biological patterns. **a**, Basic information about the test dataset (details in Methods). **b**, UMAP projections colored by species. **c**, Runtime and Maximum memory usage of each module in RamEx. **d**, Total runtime and maximum memory usage with data volume. **e**, UMAP projections colored by the multi-level phenotype analysis with varying clustering resolution (top), with detailed community structures shown in the bottom projections. **f ~ i**, UMAP projections colored by intensity or ratio values, representing specific Raman markers for ABA, HP, MAB and MTB, respectively

To evaluate RamEx’s ability to resolve inter-species phenotypic divergence at scale, we analyzed an extensive dataset comprising ~ 270,000 spectra from 344 strains across four microbial species. This dataset, encompassing over a thousand experimental batches (Fig. 5a, b), presents significant challenges in terms of size, and intrinsic complexity. To handle such data, RamEx incorporates optimized sparse matrix representations, parallel computing frameworks (GPU/CPU), and C + + code optimizations. These optimizations enabled the processing of the entire 270,000-spectrum dataset within one hour with modest memory overhead (Fig. 5c), exhibiting sub-linear scaling characteristics that allow for expansion to even larger datasets [73] (e.g., ~ 800,000 spectra; Supplementary Fig. 14, 15).

We first assessed the impact of quality control (QC) in ensuring data integrity. Without QC, outliers and low-quality spectra severely affect the downstream analyses, skewing principal components and masking genuine biological variations (Supplementary Fig. 16). Specifically, within the Raman markers module, such noise compromised the identification of species-specific markers. Among the evaluated QC methods, RamEx’s ICOD module excelled by effectively retaining informative spectra while excluding aberrant data, achieving a 99.87% prediction accuracy in downstream classification (Supplementary Fig. 17a). In contrast, overly stringent methods (e.g., Hotelling’s T^2^, PC-MCD) retained only about 60% of the data, causing significant resource wastage and potential loss of valuable biological information (Supplementary Fig. 17b). Conversely, distance-based methods failed to filter noise efficiently, extending model training times due to slower SVM convergence (Supplementary Fig. 17c). Thus, by integrating ICOD, RamEx ensures both accurate and computationally efficient data analysis.

The robustness of this pipeline was further validated through three independent tenfold cross-validations, which consistently yielded high accuracy across all species, indicating strong reproducibility (Supplementary Fig. 17 d). And impacts from class imbalance (as one of the practical challenges in real-word) were also examined by downsampling to reorganize the datset, which revealed that increased disparities in sample sizes could impact stability, particularly for minority classes, highlighting the need for balanced experimental designs (Supplementary Fig. 17e). Additionally, the resolution limits of the platform were probed. While RamEx was highly effective for general profiling, classification accuracy among closely related probiotic species dropped to approximately 76% (Supplementary Fig. 17f). This suggests that for high-resolution taxonomic classification of genetically similar strains, RamEx may benefit from integration with deeper learning architectures.

Leveraging this high-quality, large-scale dataset, RamEx revealed multi-scale biological insights ranging from phylogenetic relationships to molecular composition. First, RamEx reconstructed the phenotypic landscape of the community, capturing the hierarchical structure of microbial diversity (Fig. 5e). By varying the clustering resolution, we generated a multi-level visualization where cell affiliations align with taxonomic boundaries (Fig. 5e, top). The relative thickness of band segments in this visualization correlates with population abundance, revealing phylogenetic relationships among species. RamEx’s hierarchical clustering allow users to navigate from broad overviews to detailed inspections of specific subpopulations within the dataset, demonstrating its utility for microbiome-level profiling (Fig. 5e, bottom).

Second, RamEx identified species-specific molecular signatures that reflect underlying physiological differences. The Raman markers module of RamEx, particularly using the ROC method (one-versus-rest receiver operating characteristic), identified distinct spectral bands and ratios with high classification power. Notably, *Acinetobacter baumannii* (ABA) and *Helicobacter pylori* (HP) were distinguished by higher signal intensities at 1328 cm⁻^1^ and 1580 cm⁻^1^, respectively, than other species (Fig. 5f, g). Furthermore, RamEx effectively differentiated *Mycobacterium abscessus* (MAB) from *Mycobacterium tuberculosis* (MTB) using peak ratio combinations such as ${I}_{2852}/{I}_{2952}$ and ${I}_{1000}/{I}_{1078}$ (Fig. 5h, i). These markers reflect differences in lipid-to-protein ratios and phenylalanine-ring vibrations, which likely correspond to distinct cell-wall compositions (e.g., mycolic acid structures) and stress-response mechanisms characteristic of these two mycobacterial species.

Notably, we examined the impact of Savitzky-Golay (SG) smoothing parameters and confirmed that these identified markers largely remained stable, eliminating concerns that these features might be artifacts of specific signal processing steps (Supplementary Fig. 18). To provide a comprehensive marker identification framework, RamEx also integrates other identification methods such as DAPC (Discriminant Analysis of Principal Components) and PLS-DA (Partial Least Squares Discriminant Analysis). Although these methods differ in their underlying principles, we systematically benchmarked their performance and stability using cross-validation (Supplementary Fig. 19). The results showed that ROC was the preferred method for scenarios requiring reproducible and efficient marker selection, while DAPC and PLS-DA remained valuable tools for exploring more complex, multivariate spectral patterns.

## Discussion

RamEx was developed to address critical challenges in large-scale microbial ramanome analyses, offering an integrated solutions from spectral quality control, microbial phenotype classification, and advanced modeling across diverse biological contexts (Fig. 1). Key strength of RamEx lies in three components: robust outlier detection for designed microbial Raman spectra, specialized statistical and machine learning algorithms for spectral interpretation, and computational optimizations that enhance scalability and efficiency for high-throughput workflows.

A major challenge in high-throughput microbial Raman spectroscopy is the presence of technical artifacts, which obscure microbial spectral signatures and introduce biases in downstream analyses [50]. Existing outlier detection methods often rely on empirical thresholds, limiting their adaptability in diverse microbial communities [55, 57, 59]. RamEx addresses this challenge by integrating ICOD, which leverages a peak-centric strategy and sharpening convolution to iteratively refine feature representations and identify anomalous clusters without relying on manual parameter tuning. ICOD achieved high performance on simulated datasets (F1 = 0.97) and maintained competitive accuracy on diverse real datasets (F1 = 0.74) (Figs. 2– 3). The reduction in performance on real samples reflects the fact that biological Ramanomes contain not only technical noise but also genuine biochemical variability, making the boundary between meaningful spectra and artifacts more complex. This challenge becomes most apparent in highly heterogeneous datasets, where rare but biologically meaningful subpopulations deviate from the dominant mode. To address this issue, we introduced a heterogeneity-aware framework that first assesses dataset complexity, then applies ICOD within coarse subpopulations, followed by optional expert inspection through data rescue (Methods; Supplementary Fig. 6). This combined strategy stabilizes ICOD’s performance in complex microbial communities and helps retain rare biological groups while still removing true technical artifacts. Nevertheless, ICOD has clear applicability boundaries. Because it assumes that the statistical majority of spectra represent conserved biological fingerprints, ICOD performs best in large-scale, labeled, and relatively homogeneous datasets—such as pure-culture experiments or curated spectral libraries. In highly heterogeneous communities, ICOD results should be interpreted with caution and complemented by subpopulation-aware QC and expert inspection. This limitation arises because, in highly heterogeneous datasets, true biological variation may resemble technical artifacts in magnitude or spectral pattern, making them difficult to disentangle automatically. Biological subpopulations arise from differences in physiology, environmental stress, or pigment and metabolite accumulation, whereas technical artifacts originate from measurement issues such as misfocusing, background contamination, CCD saturation or laser-induced burning, and typically show distorted baselines, non-cellular peaks or irregular noise patterns. Distinguishing these two sources of variation is essential for responsible quality control.

Beyond outlier detection, the microbial ramanome matrix exhibits distinct statistical and spectral characteristics that set it apart from other single-cell omics data. These include non-negative spectral intensities, non-linear molecular concentration-signal relationships, vibrational mode interactions, and wavelength band correlations. Additionally, systematic and random noise are inherent to Raman spectral data, further complicating computational analysis [74–76]. Such properties lead to statistical mismatches with conventional omics-optimized workflows, particularly those designed for sparse count matrices (e.g., scRNA-seq pipelines), making direct adaptation ineffective. To address these challenges, RamEx incorporates correlation-aware feature selection to handle spectral collinearity and implements non-negativity constraints in spectral decomposition, ensuring biologically meaningful spectral interpretations, thus allowing biologists to extract metabolic and phenotypic insights without requiring expertise in algorithmic fine-tuning. However, caution is warranted when interpreting these spectral fingerprints as direct metabolic profiles. The Raman spectrum of a cell is dominated by its most abundant molecules and metabolites, meaning low-abundance regulatory proteins, transcripts, or rapid dynamic processes may remain undetectable due to the spectral sensitivity and chemical specificity limits [77]. Furthermore, spectral variability can arise not only from metabolic shifts but also from potential confounding factors such as batch effects, or stress-induced cellular degradation. Consequently, while the ramanome provides a powerful indicator for the cellular state, integrating ramanome with other omics readouts, such as metabolomics, transcriptomics, or proteomics, is still recommended for a more comprehensive and multidimensional view of phenotype [78]. Such integrative approaches, despite their own computational challenges like cross-modal data fusion, represent a vital direction for future research [79].

Processing millions of spectra from diverse instruments presents significant challenges, not only in terms of computational efficiency and data management [80, 81], but also in dealing with systematic challenges such as batch effects, instrument variability, class imbalance, and biological heterogeneity. Each of these factors can introduce bias and impact the reliability of downstream analyses. RamEx addresses the demands of efficiency and scalability by integrating C++ computation with CPU + GPU parallel processing to achieve near linear scalability (Fig. 5, Methods), while also standardizing data structures and output formats to ensure reproducible workflows across various acquisition platforms. Among these challenges, batch and instrument effects remain central concerns in large-scale spectral analysis. Factors such as laser power fluctuations, detector drift, optical misalignment, and temperature changes can all introduce significant spectral variability [16]. To reduce these impacts, RamEx integrates robust preprocessing steps and provides a fixed, reproducible analytical workflow. During data import, RamEx automatically detects whether spectra originate from multiple acquisition batches—caused by either instrument changes or measurement sessions—and reports a “Reproducibility” metric, which quantitatively reflects inter-batch consistency (Supplementary Fig. 1). This metric allows users to rapidly assess batch or instrument artefacts and overall data quality. For convenience, RamEx also includes commonly used batch‑correction options, such as piecewise direct standardization (PDS), allowing users to perform basic harmonization directly within the pipeline. When the reproducibility metric falls below the recommended threshold, users are encouraged to apply more advanced or external batch correction algorithms before downstream analysis. And ongoing development of adaptive batch‑correction algorithms is currently underway as part of the next phase of RamEx.

Data imbalance and compositional similarity between species significantly impact classification accuracy in large-scale datasets. Our evaluation showed that increased data imbalance reduced macro accuracy and stability, particularly affecting minority classes (Supplementary Fig. 17e). Although large sample sizes partially mitigated imbalance effects, as evidenced by consistently high classification accuracy in repeated cross-validation of our main dataset (γ = 18.44; Supplementary Fig. 17 d), imbalance remains a concern, especially for rare microbial populations. Strategies such as class weighting, cost-sensitive learning, data augmentation (e.g., clustering-based), and stratified cross-validation can help address this challenge [82]. Furthermore, classification accuracy strongly depends on biochemical similarity among microbial species. Using a published probiotic dataset comprising closely related species of *Lactobacillus*, *Bifidobacterium*, and *Streptococcus* [70], we observed significantly reduced classification accuracy (SVM: 76.50%; linear discriminant: 67.15%; random forest: 60.90%), due to overlapping spectral patterns from similar cell wall compositions and metabolic profiles (Supplementary Fig. 17f). Future research should focus on developing and integrating advanced analytical frameworks, particularly deep learning architectures, to improve sensitivity to subtle spectral differences and enhance taxonomic resolution among closely related microbial taxa.

Looking ahead, several directions are critical for advancing Raman-based single-cell microbiology. First, there is a clear need for large-scale, pre-trained deep learning models. Trained on diverse spectral libraries, such models could provide robust, generalizable solutions for spectral denoising and, critically, instrument-agnostic normalization, which remains a major barrier to data reuse. These models could then be rapidly fine-tuned with minimal task-specific data. Second, building on recent successes in paired multi-omics, a major computational goal is the deeper integration of Ramanomes with transcriptomic or proteomic data. This requires advanced representation learning frameworks that can reliably map complex spectral phenotypes to their underlying molecular (e.g., gene regulatory) and functional states. Finally, the integration of these predictive models into real-time flow cytometry applications is essential. This would enable a shift from offline analysis to AI-guided, closed-loop experiments. For instance, implementing active learning algorithms on Raman flow cytometers could allow for the intelligent and automated sorting of rare, transient, or phenotypically novel cells, greatly accelerating functional discoveries. The successful implementation of such systems, however, will depend not only on continued advances in data analysis algorithms but also critically on parallel developments in hardware technologies, such as sorting throughput and speed.

## Conclusion

RamEx improves microbial ramanome analysis with its adaptive, threshold-free Iterative Convolutional Outlier Detection (ICOD), achieving exceptional spectral quality control (F1 > 0.97). By integrating modular workflows for phenotype clustering, metabolic-associated fingerprinting, and intra-population heterogeneity analysis, it effectively bridges key computational gaps in Raman-based microbial phenomics. Optimized for scalability, RamEx efficiently processes million-scale datasets, enabling applications from antibiotic resistance tracking to functional dynamics studies at single-cell resolution. As a freely available R package, RamEx establishes a standardized framework for reproducible, high-throughput ramanomics, positioning Raman flow cytometry as a mainstream tool in microbial systems biology.

## Methods

### Datasets

Five public datasets were used for evaluation: (*i*) “bacteria” dataset from Liu et al. [13], containing Raman spectra of 15 pathogenic bacterial isolates; (*ii*) “*Mycobacterium tuberculosis*” dataset from Mao et al., comprising single-cell Raman spectra of drug-sensitive and resistant MTB strains under various conditions; (*iii*) “probiotic” dataset from Zhang et al., containing spectra of four commercial probiotic strains collected using FlowRACS; (4) “*E. coli*” dataset from Wang et al. [23], including spectra of *E. coli* strains exposed to three antibiotics; and (*v*) “spores” dataset from wild-type, mutant, and edited strains of *Aspergillus candidus* collected via FlowRACS. Additionally, two self-collected datasets were used: a yeast dataset tracking *S. cerevisiae* during pilot-scale fermentation (16 time points, 0–100 h), and a large-scale bacterial dataset comprising four species (*A. baumannii*, *H. pylori*, *M. abscessus*, and *M. tuberculosis*) across 344 strains under various experimental conditions. Both datasets were collected using a FlowRACS instrument (Qingdao Single-cell Biotech, CN, 532nm laser, 100 mW power, 1 s integration, 100 × objective).

### Data simulation

Simulated Ramans spectra were generated by Gaussian process regression (GPR) using the real Raman spectral data of *Escherichia coli* ATCC 25922 as reference [23]. Single-cell heterogeneity was simulated using radial basis function and white noise kernels, while instrumental variations were modeled by adding random Gaussian noise and polynomial baselines (orders 1–4, coefficients ± 0.01). Four types of spectral anomalies [55] were simulated using homemade scripts: (*i*) Laser-induced burning effect: Signal overflow with sudden intensity spikes (60,000 counts); (*ii*) Optical defocusing: Selective enhancement/attenuation of spectral regions identified by the bubble method; (*iii*) Device artifact: Random addition of 20 overlapping peaks (width 200–1,000 cm⁻^1^, intensity 100–1,000 counts); (*iv*) Empty acquisition: Substrate signals modeled with a 700 cm⁻^1^ Gaussian peak and instrumental noise. The four types of anomalies were randomly introduced into the generated spectra, thus creating 19 anomalous datasets with anomaly proportions ranging from 5 to 95%. Five replicates of such datasets were generated for downstream analysis.

### Batch effect detection and correction

RamEx automatically evaluates batch consistency among imported spectra based on wavenumber alignment, allowing the software to estimate how many distinct batches are present in the dataset. For each batch pair, RamEx computes a Reproducibility index that quantitatively reflects inter‑batch distinction. Users can then optionally apply the built‑in Piecewise Direct Standardization (PDS) algorithm to correct systematic inter‑batch differences. After correction, the reproducibility index is recalculated, and users can assess the reduction of batch effects through low‑dimensional visualization (e.g., t‑SNE or UMAP). If batch harmonization remains unsatisfactory, the corrected spectral matrix exported by RamEx can be further processed using external or advanced batch‑effect removal methods.

### Iterative Convolutional Outlier Detection (ICOD)

The ICOD is specifically designed to identify diverse outliers in ramanome data, leveraging an iterative, unsupervised framework to enhance robustness and precision in high-dimensional spectral data. The method integrates peak detection via a modified bubble algorithm, fuzzy convolution, and clustering analysis to systematically isolate anomalous signals. The main workflow includes:

1. Initialization and Matrix Preparation. All Raman spectra are preprocessed using the default RamEx workflow. Spectra with missing values (NA) are excluded; all others are initially labeled as valid.
2. Peak Extraction with Modified Bubble Method. Peaks are detected with a bubble baseline algorithm applied to the average spectrum of the currently valid samples. The minimum bubble width is set to 100 cm⁻^1^ to avoid capturing minor noise features. During the baseline estimation, the algorithm records the regions where bubbles interact with the spectrum, and the highest signal point within each region is identified as the peak position. This reduces the original high-dimensional dataset (${X}_{n\times m}$) to a peak-feature matrix (${X}^{\prime}_{n\times p}$ with $p\ll m$), which isolates critical spectral features while discarding irrelevant noise and redundancy, ensuring the preservation of essential information for downstream analysis.
3. Local Outliers. Local anomalies are identified in the peak-feature matrix using the quartile method. For each peak $j$, an observation ${x}_{ij}$ is classified as a local outlier if it satisfies the conditions ${x}_{ij}<{Q1}_{j}-1.5\times {IQR}_{j}$ or ${x}_{ij}>{Q3}_{j}+1.5\times {IQR}_{j}$, where ${Q1}_{j}$ and ${Q3}_{j}$ represent the first and third quartiles, respectively, and ${IQR}_{j}$ is the interquartile range. This results in a Boolean matrix ${B}_{n\times p}$, where ${B}_{ij}=1$ if ${x}_{ij}$ is an outlier and ${B}_{ij}=0$ otherwise.
4. Global Outliers. To enhance the robustness of clustering, mean convolution filtering is applied to the Boolean matrix, reducing the influence of isolated local anomalies and improving the consistency. The Boolean matrix is then subjected to k-means clustering with $k=2$, separating spectra into normal and anomalous categories.
5. Iteration and Stopping Criteria. Steps 2–4 are repeated, updating the global outlier list at each iteration, adjusting the probability of each spectrum being classified as an outlier. To avoid progressive over-rejection, spectra flagged as abnormal are masked from statistical calculations (e.g., peak detection, quantile computation) but remain in the clustering step throughout all iterations. This iterative process terminates when the global outlier list no longer changes between iterations. Optionally, if a threshold for the proportion of variable (anomalous) peaks is specified, the algorithm will then evaluate whether the proportion of anomalous peaks in the retained (normal) spectra exceeds the predefined threshold (e.g., 50%). If so, a new round of ICOD is performed on the remaining normal spectra for further refinement.

### Heterogeneity-aware quality control

To ensure that ICOD is applied appropriately in datasets with varying levels of intrinsic diversity, RamEx incorporates a heterogeneity-aware workflow consisting of three steps.

1. Assessment of dataset complexity using Heterogeneity Index (HI). RamEx first evaluates the presence of latent subpopulation structure by performing a low-resolution Louvain clustering (resolution = 0.01). Two complementary quantities are then computed: (i) the entropy of the cluster-size distribution, which reflects how evenly spectral subgroups populate the dataset, and (ii) the average inter-cluster distance, which measures how well these subgroups are separated in feature space. HI is defined as the product of these two quantities, yielding a value between 0 and 1. Low HI values indicate a single dominant spectral mode suitable for global ICOD, whereas high HI values (> 0.8) indicate multiple partially segregated spectral modes, which requires stratified ICOD.
2. ICOD computation and high-resolution structural assessment. For homogeneous datasets (low HI), ICOD is applied directly to the full dataset. For heterogeneous datasets (high HI), ICOD is applied within each subpopulation independently to avoid misclassifying biologically valid but distinct groups. Then RamEx performs high-resolution Louvain clustering (resolution = 1) on provisionally rejected spectra to characterize their internal structure.
3. Secondary expert-guided inspection for data rescue. RamEx visualizes high-resolution clusters from rejected spectra to facilitate expert review, where coherent biological subpopulations incorrectly deleted by ICOD can be reinstated from the rejected matrix (data rescue). This optional refinement is particularly valuable for datasets containing rare pigment-rich or stress-responsive subpopulations.

### Performance evaluation of ICOD

The performance of ICOD and other outlier detection methods was evaluated using both simulated and real datasets. For the simulated datasets, the ground truth labels were generated alongside the random definition of anomalous spectra. For the real datasets, quality labels were manually assigned to each spectrum based on expert evaluation. To minimize bias from preprocessing, all data used for evaluation were processed using the default preprocessing pipeline of RamEx prior to outlier detection.

The evaluated methods included ICOD, the signal-to-noise ratio (SNR) method, the principal components—minimum covariance determinant (PC-MCD), the Hotelling’s T^2^ method, and the Euclidean distance method. The accuracy of quality control was assessed using three metrics: precision, recall, and F1 score. These metrics are defined as follows: $Precision=\frac{TP}{TP+FP}, Recall=\frac{TP}{TP+FN}$, where True Positives (TP) represent spectra correctly identified as anomalous, False Positives (FP) represent spectra incorrectly classified as anomalous, and False Negatives (FN) represent anomalous spectra that were incorrectly classified as normal (undetected anomalies). F1 score is the harmonic mean of the precision and recall and is calculated as $F1 score=2\times \frac{precision \times recall}{precision+recall}$. For the real datasets, the predicted labels generated by each of the five outlier detection methods were compared against the manually assigned ground truth labels, and the above evaluation metrics were calculated. For the simulated datasets, the metrics were computed for five parallel datasets with varying proportions of anomalies, providing a comprehensive assessment of each method’s performance under different conditions.

### Raman spectral preprocessing

The raw Raman spectra was preprocessed using the default pipeline in RamEx, including cosmic ray removal, spectral smoothing, baseline correction, and normalization. These steps were applied sequentially to ensure the quality and consistency of the spectral data. Cosmic ray removal was performed using a two-dimensional convolution-based spike detection method with a sharpening convolution kernel, where the center pixel value was set to 33. Signals in the identified cosmic ray regions were replaced with the corresponding intensities from adjacent spectra to minimizing distortion. Smoothing was conducted using the Savitzky-Golay method with a window size of 11 and a 5th-order polynomial fit, effectively reducing high-frequency noise while preserving spectral features. Baseline correction was then applied to remove low-frequency spectral noise using partitioned polynomial fitting. Specifically, a first-order polynomial was fitted to the 500–1800 cm⁻^1^ region, while a sixth-order polynomial was applied to the 1800–3050 cm⁻^1^ region to account for the varying baseline characteristics across the spectrum. Each spectrum was normalized to the maximum intensity value within the CH stretching region (2750–3050 cm⁻^1^), ensuring comparability across spectra.

### Classification

A support vector machine (SVM) with linear kernel was employed for classification, using a 70:30 train-test split. Each dataset was randomly divided into 70% for model training and 30% for testing to enable a fair performance assessment and to evaluate how different quality‑control methods influenced classification accuracy. To further confirm the robustness of the classification module with ICOD, three independent tenfold cross‑validations were performed. To address the computational complexity of $O({d}_{L}{N}_{S})$, where ${N}_{S}$ is the number of support vectors and ${d}_{L}$ is the dimensionality, PCA was applied to reduce the spectral data to 20 principal components before SVM training. Multi-class classification was handled using the “one-against-one” strategy, with test data projected into the same PCA space.

### Marker identification

Raman markers were identified as individual bands or band pairs strongly associated with ground truth labels. For continuous labels, Pearson correlation coefficients were used, while for categorical labels, the area under the ROC curve (AUC) in a “one-versus-rest” framework quantified discriminative ability. Markers were selected when their correlations or AUC scores exceeded predefined thresholds. To overcome the computational complexity of evaluating band combinations (${C}_{n}^{2}$), the analysis was accelerated using C++ implementation via “Rcpp” with efficient memory management and multi-core parallelization. While RamEx defaults to single-band analysis, this study evaluated both single-band and paired-band combinations for comprehensive marker identification.

### Feature reduction and phenotype analysis

Nonlinear dimensionality reduction methods, such as Uniform Manifold Approximation and Projection (UMAP) and t-distributed Stochastic Neighbor Embedding (t-SNE), were employed for the visualization of ramanome datasets. To ensure computational efficiency, the spectral matrix was first subjected to principal component analysis (PCA) for preliminary dimensionality reduction. Additionally, the visualization process was accelerated using multithreaded computation. Cellular phenotypes within the ramanome were identified using the Louvain community detection method based on shared nearest neighbor (SNN). After computing k-nearest neighbors and constructing an SNN graph using cosine similarity, clusters were identified through modularity optimization. Small clusters (< 1%) were merged with their nearest communities, and for datasets exceeding 10,000 samples, the SNN graph was constructed based on sparse distance matrix. Multiple resolution parameters (0.01–0.2) were applied to generate a hierarchical phenotypic structure, with results visualized using UMAP.

### Spectral decomposition

To decompose the dataset into its underlying components and corresponding concentration profiles, the Multivariate Curve Resolution-Alternating Least Squares (MCR-ALS) algorithm was employed. The algorithm iteratively optimizes the decomposition of the dataset $X$ into two matrices, as described by the following equation:

$$X=C\bullet {S}^{T}+E$$

where $C$ represents the concentration matrix, $S$ is the spectral matrix of components, and $E$ denotes the residual matrix. Nonnegativity constraints were imposed on both concentration and spectral components. Additionally, an angle constraint was applied to minimize collinearity between components during the optimization process.

### Intra-ramanome analysis

Intra-ramanome correlation analysis (IRCA) was conducted to observe the heterogeneity within the ramanome and to elucidate intracellular metabolite conversions. For detailed methodological descriptions, refer to He et al. [66]. In this study, global and local intra-ramanome correlation networks (global-IRCNs and local-IRCNs) were constructed at different fermentation time points, with thresholds of $p<0.05$ and $\rho <-0.6$. The global-IRCN considers variations across all Raman bands, providing a comprehensive overview of the ramanome’s dynamic changes, whereas the local-IRCN focuses on specific bands of interest that represent key biomolecular components. For example, bands at 781 and 1575 cm^−1^ correspond to nucleic acids; 847, 886, 915, and 1120 cm^−1^ to polysaccharides; 995, 1148, 1330 and 2920 cm^−1^ to proteins; and 1295, 1438, 1591, 1655, 1738, 2842, and 2900 cm^−1^ to lipids. (Supplementary Table 4).

### Memory usage and time-consuming tests

Tests on a large dataset containing Raman spectra from four bacterial species were used. Random subsets of 10,000 to 270,000 spectra were sampled and analyzed following the RamEx workflow, including six main steps: data import, quality control, spectral preprocessing, feature reduction, Raman marker identification, and classification. For classification, the dataset was split into training and testing sets with a 70:30 ratio. The time consumption of each step was recorded using R’s built-in “Sys.time()” function to measure the intermediate processing times. Peak memory usage during the analysis was monitored and measured using the “Rprof” function to capture the maximum memory allocation. All analyses were performed on a CentOS Linux 7 server with an Intel Xeon E7-4820 v4 processor (80 cores, 2.00 GHz), 575 GB RAM, using R 4.3.0 and OpenBLAS 0.3.3 with LAPACK 3.8.0.

## Supplementary Information

Supplementary Material 1.

Supplementary Material 2.

## Data Availability

All datasets that support the findings of this study have been deposited in Science Data Bank and can be accessed from https://www.scidb.cn/en/s/ju6VNf.

## Abbreviations

ABA: *Acinetobacter baumannii*
ALS: Asymmetric Least Squares
AUC: Area Under the Curve
GMM: Gaussian Mixture Model
GPU: Graphics Processing Unit
HP: *Helicobacter pylori*
ICOD: Iterative Convolutional Outlier Detection
ICA: Independent Component Analysis
IRCA: Intra-Ramanome Correlation Analysis
IRCN: Intra-Ramanome Correlation Network
KNN: K-Nearest Neighbor
LDA: Linear Discriminant Analysis
MAB: *Mycobacterium abscessus*
MCR-ALS: Multivariate Curve Resolution–Alternating Least Squares
MLR: Multiple Linear Regression
MTB: *Mycobacterium tuberculosis*
NMF: Non-negative Matrix Factorization
PC-MCD: Principal Components–Minimum Covariance Determinant
PCA: Principal Component Analysis
PLS: Partial Least Squares Regression
QC: Quality Control
RBCS: Raman Barcode of Cellular-response to Stresses
ROC: Receiver Operating Characteristic
SCRS: Single-Cell Raman Spectroscopy
SNIP: Statistics-Sensitive Non-Linear Iterative Peak-Clipping
SNR: Signal-to-Noise Ratio
SVM: Supporting Vector Machine
t-SNE: T-distributed Stochastic Neighbor Embedding
TAGs: Triacylglycerides
UMAP: Uniform Manifold Approximation and Projection

## References

[1] Wang D, He P, Wang Z, Li G, Majed N, Gu AZ. Advances in single cell raman spectroscopy technologies for biological and environmental applications. Curr Opin Biotechnol. 2020;64:218–29. 32688195 10.1016/j.copbio.2020.06.011

[2] Shipp DW, F Sinjab, I Notingher. Raman spectroscopy: techniques and applications in the life sciences. Adv Opt Photonics. 2017;9(2):315.

[3] Fernandez-Galiana A, Bibikova O, Pedersen SV, Stevens MM. Fundamentals and applications of raman-based techniques for the design and development of active biomedical materials. Adv Mater. 2024;36(43):e2210807. 37001970 10.1002/adma.202210807

[4] Pliss A, Kuzmin AN, Lita A, Kumar R, Celiku O, Atilla-Gokcumen GE, et al. A single-organelle optical omics platform for cell science and biomarker discovery. Anal Chem. 2021;93(23):8281–90. 34048235 10.1021/acs.analchem.1c01131 PMC8865453

[5] Ali A, S Davidson, E Fraenkel, I Gilmore, T Hankemeier, JA Kirwan, AN Lane, I Lanekoff, M Larion, LI McCall, M Murphy, JV Sweedler, C Zhu. Single cell metabolism: current and future trends. Metabolomics. 2022;18(10):77. 10.1007/s11306-022-01934-3 PMC10063251 36181583

[6] Ho CS, Jean N, Hogan CA, Blackmon L, Jeffrey SS, Holodniy M, et al. Rapid identification of pathogenic bacteria using Raman spectroscopy and deep learning. Nat Commun. 2019;10(1):4927. 31666527 10.1038/s41467-019-12898-9 PMC6960993

[7] Heidari Baladehi M, Hekmatara M, He Y, Bhaskar Y, Wang Z, Liu L, et al. Culture-free identification and metabolic profiling of microalgal single cells via ensemble learning of ramanomes. Anal Chem. 2021;93(25):8872–80. 34142549 10.1021/acs.analchem.1c01015

[8] Kloss S, Kampe B, Sachse S, Rosch P, Straube E, Pfister W, et al. Culture independent Raman spectroscopic identification of urinary tract infection pathogens: a proof of principle study. Anal Chem. 2013;85(20):9610–6. 24010860 10.1021/ac401806f

[9] Moudrikova S, Sadowsky A, Metzger S, Nedbal L, Mettler-Altmann T, Mojzes P. Quantification of polyphosphate in microalgae by Raman microscopy and by a reference enzymatic assay. Anal Chem. 2017;89(22):12006–13. 29099580 10.1021/acs.analchem.7b02393

[10] Wang T, Ji Y, Wang Y, Jia J, Li J, Huang S, et al. Quantitative dynamics of triacylglycerol accumulation in microalgae populations at single-cell resolution revealed by Raman microspectroscopy. Biotechnol Biofuels. 2014;7:58. 24716544 10.1186/1754-6834-7-58 PMC4022372

[11] He Y, Zhang P, Huang S, Wang T, Ji Y, Xu J. Label-free, simultaneous quantification of starch, protein and triacylglycerol in single microalgal cells. Biotechnol Biofuels. 2017;10:275. 29177009 10.1186/s13068-017-0967-x PMC5693592

[12] Zhang Y, Li HZ, Breed M, Tang Z, Cui L, Zhu YG, et al. Soil warming increases the active antibiotic resistome in the gut of invasive giant African snails. Microbiome. 2025;13(1):42. 39915809 10.1186/s40168-025-02044-7 PMC11800439

[13] Liu M, Zhu P, Zhang L, Gong Y, Wang C, Sun L, et al. Single-cell identification, drug susceptibility test, and whole-genome sequencing of *Helicobacter pylori* directly from gastric biopsy by clinical antimicrobial susceptibility test ramanometry. Clin Chem. 2022;68(8):1064–74. 35714147 10.1093/clinchem/hvac082

[14] Li HZ, Peng J, Yang K, Zhang Y, Chen QL, Zhu YG, et al. Single-cell exploration of active phosphate-solubilizing bacteria across diverse soil matrices for sustainable phosphorus management. Nat Food. 2024;5(8):673–83. 39103543 10.1038/s43016-024-01024-8

[15] Cui L, Xin Y, Yang K, Li H, Tan F, Zhang Y, et al. Live tracking metabolic networks and physiological responses within microbial assemblages at single-cell level. PNAS Nexus. 2023;2(3):pgad006. 36896131 10.1093/pnasnexus/pgad006 PMC9991459

[16] Zhu XQ, Xu T, Lin QY, Duan YX. Technical development of Raman spectroscopy: from instrumental to advanced combined technologies. Appl Spectrosc Rev. 2014;49(1):64–82.

[17] Pan T, TY Gao, XH Fan, ML Sa, XJ Yang, JN Xu, X Xu, M Ma, R Wang, Y Zhang, W Ye, YP Shi, HX Zhang, ZC Zeng. Development of a cost-effective confocal Raman microscopy with high sensitivity. Talanta 2025;281:126754. 10.1016/j.talanta.2024.126754 39241646

[18] Li MQ, Ashok PC, Dholakia K, Huang WE. Raman-activated cell counting for profiling carbon dioxide fixing microorganisms. J Phys Chem A. 2012;116(25):6560–3. 22320431 10.1021/jp212619n

[19] Wang X, Ren L, Su Y, Ji Y, Liu Y, Li C, et al. Raman-activated droplet sorting (RADS) for label-free high-throughput screening of microalgal single-cells. Anal Chem. 2017;89(22):12569–77. 29099582 10.1021/acs.analchem.7b03884

[20] Wang X, Xin Y, Ren L, Sun Z, Zhu P, Ji Y, et al. Positive dielectrophoresis-based raman-activated droplet sorting for culture-free and label-free screening of enzyme function *in vivo*. Sci Adv. 2020;6(32):eabb3521. 32821836 10.1126/sciadv.abb3521 PMC7413728

[21] Yan S, Qiu J, Guo L, Li D, Xu D, Liu Q. Development overview of raman-activated cell sorting devoted to bacterial detection at single-cell level. Appl Microbiol Biotechnol. 2021;105(4):1315–31. 33481066 10.1007/s00253-020-11081-1

[22] Lindley M, T Kubo, S Devineau, M Li, K Fujita. Time delay integration Raman flow cytometry. SPIE BiOS. 2024. High-Speed Biomedical Imaging and Spectroscopy IX, PC128530K.

[23] Wang X, Ren L, Diao Z, He Y, Zhang J, Liu M, et al. Robust spontaneous raman flow cytometry for single-cell metabolic phenome profiling via pDEP-DLD-RFC. Adv Sci (Weinh). 2023;10(16):e2207497. 36871147 10.1002/advs.202207497 PMC10238217

[24] Zhang C, Huang KC, Rajwa B, Li JJ, Yang SQ, Lin HN, et al. Stimulated raman scattering flow cytometry for label-free single-particle analysis. Optica. 2017;4(1):103–9. 39238893 10.1364/optica.4.000103 PMC11375991

[25] Schie IW, Huser T. Methods and applications of raman microspectroscopy to single-cell analysis. Appl Spectrosc. 2013;67(8):813–28. 23876720 10.1366/12-06971

[26] Garcia-Timermans C, Rubbens P, Heyse J, Kerckhof FM, Props R, Skirtach AG, et al. Discriminating bacterial phenotypes at the population and single-cell level: a comparison of flow cytometry and raman spectroscopy fingerprinting. Cytometry A. 2020;97(7):713–26. 31889414 10.1002/cyto.a.23952

[27] Kukolj T, J Lazarevic, A Borojevic, U Ralevic, D Vujic, A Jaukovic, N Lazarevic, D Bugarski. A single-cell Raman spectroscopy analysis of bone marrow mesenchymal stem/stromal cells to identify inter-individual diversity. Int J Mol Sci. 2022;23(9):4915. 10.3390/ijms23094915 PMC9103070 35563306

[28] Lee KS, Landry Z, Athar A, Alcolombri U, Pramoj Na Ayutthaya P, Berry D, et al. MicrobioRaman: an open-access web repository for microbiological Raman spectroscopy data. Nat Microbiol. 2024;9(5):1152–6. 38714759 10.1038/s41564-024-01656-3

[29] Liu S, Moon CD, Zheng N, Huws S, Zhao S, Wang J. Opportunities and challenges of using metagenomic data to bring uncultured microbes into cultivation. Microbiome. 2022;10(1):76. 35546409 10.1186/s40168-022-01272-5 PMC9097414

[30] Wang Y, Xu J, Kong L, Li B, Li H, Huang WE, et al. Raman-activated sorting of antibiotic-resistant bacteria in human gut microbiota. Environ Microbiol. 2020;22(7):2613–24. 32114713 10.1111/1462-2920.14962 PMC7383503

[31] Reisner LA, Cao A, Pandya AK. An integrated software system for processing, analyzing, and classifying raman spectra. Chemometr Intell Lab Syst. 2011;105(1):83–90.

[32] Song D, Chen Y, Li J, Wang H, Ning T, Wang S. A graphical user interface (NWUSA) for Raman spectral processing, analysis and feature recognition. J Biophotonics. 2021;14(5):e202000456. 33547854 10.1002/jbio.202000456

[33] Felten J, Hall H, Jaumot J, Tauler R, de Juan A, Gorzsas A. Vibrational spectroscopic image analysis of biological material using multivariate curve resolution-alternating least squares (MCR-ALS). Nat Protoc. 2015;10(2):217–40. 25569330 10.1038/nprot.2015.008

[34] Gibb S, Strimmer K. MALDIquant: a versatile R package for the analysis of mass spectrometry data. Bioinformatics. 2012;28(17):2270–1. 22796955 10.1093/bioinformatics/bts447

[35] Georgiev D, Pedersen SV, Xie R, Fernandez-Galiana A, Stevens MM, Barahona M. Ramanspy: an open-source Python package for integrative Raman spectroscopy data analysis. Anal Chem. 2024;96(21):8492–500. 38747470 10.1021/acs.analchem.4c00383 PMC11140669

[36] Gao C, P Zhao, Q Fan, H Jing, R Dang, W Sun, Y Feng, B Hu, Q Wang. Deep neural network: as the novel pipelines in multiple preprocessing for Raman spectroscopy. Spectrochim Acta A: Mol Biomol Spectrosc 2023;302:123086. 10.1016/j.saa.2023.123086 37451210

[37] Wahl J, Sjödahl M, Ramser K. Single-step preprocessing of Raman spectra using convolutional neural networks. Appl Spectrosc. 2020;74(4):427–38. 31961223 10.1177/0003702819888949

[38] Chen T, Son Y, Park A, Baek SJ. Baseline correction using a deep-learning model combining ResNet and UNet. Analyst. 2022;147(19):4285–92. 36000247 10.1039/d2an00868h

[39] Jermyn M, J Desroches, J Mercier, K St-Arnaud, MC Guiot, K Petrecca, F Leblond. Neural networks improve brain cancer detection with Raman spectroscopy in the presence of light artifacts. SPIE BiOS. 2016;9690:40. SPIE. 10.1117/1.JBO.21.9.094002 27604560

[40] Satija R, Farrell JA, Gennert D, Schier AF, Regev A. Spatial reconstruction of single-cell gene expression data. Nat Biotechnol. 2015;33(5):495-U206. 25867923 10.1038/nbt.3192 PMC4430369

[41] Zhao E, Stone MR, Ren X, Guenthoer J, Smythe KS, Pulliam T, et al. Spatial transcriptomics at subspot resolution with BayesSpace. Nat Biotechnol. 2021;39(11):1375–84. 34083791 10.1038/s41587-021-00935-2 PMC8763026

[42] Trapnell C, Cacchiarelli D, Grimsby J, Pokharel P, Li S, Morse M, et al. The dynamics and regulators of cell fate decisions are revealed by pseudotemporal ordering of single cells. Nat Biotechnol. 2014;32(4):381–6. 24658644 10.1038/nbt.2859 PMC4122333

[43] Sigle M, Rohlfing AK, Kenny M, Scheuermann S, Sun N, Graessner U, et al. Translating genomic tools to Raman spectroscopy analysis enables high-dimensional tissue characterization on molecular resolution. Nat Commun. 2023;14(1):5799. 37726278 10.1038/s41467-023-41417-0 PMC10509269

[44] Movasaghi Z, Rehman S, Rehman IU. Raman spectroscopy of biological tissues. Appl Spectrosc Rev. 2007;42(5):493–541.

[45] Praus P, Stepanek J. Statistical signal-processing in Raman-spectroscopy of biological samples. SPIE Proc. 1991;1403:76–84.

[46] Jiang R, T Sun, D Song, JJ Li. Statistics or biology: the zero-inflation controversy about scRNA-seq data. Genome Biol. 2022;23(1):31. 10.1186/s13059-022-02601-5 PMC8783472 35063006

[47] Dadaneh SZ, P de Figueiredo, SH Sze, M Zhou, X Qian. Bayesian gamma-negative binomial modeling of single-cell RNA sequencing data. BMC Genomics. 2020:21(S9):585. 10.1186/s12864-020-06938-8 PMC7487589 32900358

[48] Artemyev DN, AA Shatskaya. Study of spurious optical signals in a fiber-optic Raman spectroscopy system. Opt Laser Technol. 2022;152: 108184.

[49] Bowie BT, DB Chase, IR Lewis, PR Griffiths. Anomalies and artifacts in Raman spectroscopy. In: Handbook of vibrational spectroscopy. 2001.

[50] Vulchi RT, V Morgunov, R Junjuri, T Bocklitz. Artifacts and anomalies in Raman spectroscopy: a review on origins and correction procedures. Molecules. 2024;29(19):4748. 10.3390/molecules29194748 PMC11478279 39407680

[51] Massie C, Chen K, Berger AJ. Calibration technique for suppressing residual etalon artifacts in slit-averaged Raman spectroscopy. Appl Spectrosc. 2021;76(2):255–61. 34596460 10.1177/00037028211046643 PMC8831449

[52] Pence I, Mahadevan-Jansen A. Clinical instrumentation and applications of raman spectroscopy. Chem Soc Rev. 2016;45(7):1958–79. 26999370 10.1039/c5cs00581g PMC4854574

[53] Kniese J, AM Race, H Schmidt. Classification of cereal flour species using Raman spectroscopy in combination with spectra quality control and multivariate statistical analysis. J Cereal Sci. 2021;101:103299.

[54] Dallaire F, Picot F, Tremblay JP, Sheehy G, Lemoine E, Agarwal R, et al. Quantitative spectral quality assessment technique validated using intraoperative in vivo raman spectroscopy measurements. J Biomed Opt. 2020;25(4):1–8. 32319263 10.1117/1.JBO.25.4.040501 PMC7171512

[55] Guo S, J Popp, T Bocklitz. Chemometric analysis in Raman spectroscopy from experimental design to machine learning-based modeling. Nat Protoc. 2021;16:5426–59. https://www.nature.com/articles/s41596-021-00620-3. 10.1038/s41596-021-00620-3 34741152

[56] Xu JB, Yi XF, Jin GL, Peng D, Fan GY, Xu XG, et al. High-speed diagnosis of bacterial pathogens at the single cell level by Raman microspectroscopy with machine learning filters and denoising autoencoders. ACS Chem Biol. 2022;17(2):376–85. 35026119 10.1021/acschembio.1c00834

[57] Yang XN, Wang ZJ, Zi XM. Thresholding-based outlier detection for high-dimensional data. J Stat Comput Simul. 2018;88(11):2170–84.

[58] Schulze HG, Rangan S, Piret JM, Blades MW, Turner RFB. Developing fully automated quality control methods for preprocessing Raman spectra of biomedical and biological samples. Appl Spectrosc. 2018;72(9):1322–40. 29855196 10.1177/0003702818778031

[59] Brownfield B, Kalivas JH. Consensus outlier detection using sum of ranking differences of common and new outlier measures without tuning parameter selections. Anal Chem. 2017;89(9):5087–94. 28367620 10.1021/acs.analchem.7b00637

[60] Qi Y, D Hu, Y Jiang, Z Wu, M Zheng, EX Chen, Y Liang, MA Sadi, K Zhang, YP Chen. Recent progresses in machine learning assisted raman spectroscopy. Adv Opt Mater. 2023:11(14):2203104.

[61] Eilers P, H Boelens. Baseline correction with asymmetric least squares smoothing. Leiden. 2005.

[62] Schaible GA, Cliff JB, Crandall JA, Bougoure JJ, Mathuri MN, Sessions AL, et al. Comparing raman and nanosims for heavy water labeling of single cells. Microbiol Spectr. 2025;13(7):e0165924. 40445204 10.1128/spectrum.01659-24 PMC12211084

[63] Ryan CG, Clayton E, Griffin WL, Sie SH, Cousens DR. Snip, a statistics-sensitive background treatment for the quantitative analysis of PIXE spectra in geoscience applications. Nucl Instrum Methods Phys Res Sect B Beam Interact Mater Atoms. 1988;34(3):396–402.

[64] Berry D, Mader E, Lee TK, Woebken D, Wang Y, Zhu D, et al. Tracking heavy water (D2O) incorporation for identifying and sorting active microbial cells. Proc Natl Acad Sci U S A. 2015;112(2):E194-203. 25550518 10.1073/pnas.1420406112 PMC4299247

[65] Teng L, X Wang, XJ Wang, HL Gou, LH Ren, TT Wang, Y Wang, YT Ji, WE Huang, J Xu. Label-free, rapid and quantitative phenotyping of stress response in E. coli via ramanome. Sci Rep. 2016;6:34359. 10.1038/srep34359 PMC5069462 27756907

[66] He Y, Huang S, Zhang P, Ji Y, Xu J. Intra-ramanome correlation analysis unveils metabolite conversion network from an isogenic population of cells. MBio. 2021;12(4):e0147021. 34465024 10.1128/mBio.01470-21 PMC8406334

[67] Sheehy G, Picot F, Dallaire F, Ember K, Nguyen T, Petrecca K, et al. Open-sourced raman spectroscopy data processing package implementing a baseline removal algorithm validated from multiple datasets acquired in human tissue and biofluids. J Biomed Opt. 2023;28(2):025002. 36825245 10.1117/1.JBO.28.2.025002 PMC9941747

[68] Alfaro JL, Ortega JF. Robust Hotelling’s T2 control charts under non-normality: the case of t-Student distribution. J Stat Comput Simul. 2012;82(10):1437–47.

[69] Rousseeuw PJ, Van Driessen K. A fast algorithm for the minimum covariance determinant estimator. Technometrics. 1999;41(3):212–23.

[70] Zhang J, Ren L, Zhang L, Gong Y, Xu T, Wang X, et al. Single-cell rapid identification, in situ viability and vitality profiling, and genome-based source-tracking for probiotics products. Imeta. 2023;2(3):e117. 38867931 10.1002/imt2.117 PMC10989769

[71] Hsu CC, Xu J, Brinkhof B, Wang H, Cui Z, Huang WE, et al. A single-cell raman-based platform to identify developmental stages of human pluripotent stem cell-derived neurons. Proc Natl Acad Sci U S A. 2020;117(31):18412–23. 32694205 10.1073/pnas.2001906117 PMC7414136

[72] Huang YS, Karashima T, Yamamoto M, Hamaguchi HO. Molecular-level investigation of the structure, transformation, and bioactivity of single living fission yeast cells by time- and space-resolved raman spectroscopy. Biochemistry. 2005;44(30):10009–19. 16042377 10.1021/bi050179w

[73] Hill IE, Boyd M, Milligan K, Jenkins CA, Sorensen A, Jirasek A, et al. Understanding radiation response and cell cycle variation in brain tumour cells using raman spectroscopy. Analyst. 2023;148(11):2594–608. 37166147 10.1039/d3an00121k PMC10228487

[74] Jones RR, Hooper DC, Zhang L, Wolverson D, Valev VK. Raman techniques: fundamentals and frontiers. Nanoscale Res Lett. 2019;14(1):231. 31300945 10.1186/s11671-019-3039-2 PMC6626094

[75] Arora AK, Umadevi V. Instrumental distortions of raman lines. Appl Spectrosc. 1982;36(4):424–7.

[76] Schulze HG, Rangan S, Vardaki MZ, Blades MW, Turner RFB, Piret JM. Critical evaluation of spectral resolution enhancement methods for Raman hyperspectra. Appl Spectrosc. 2022;76(1):61–80. 34933587 10.1177/00037028211061174 PMC8750138

[77] Nishiyama R, Furuya K, Tamura T, Nakao R, Peterson W, Hiramatsu K, et al. Fourier transform coherent anti-Stokes Raman scattering spectroscopy: a comprehensive review. Anal Chem. 2024;96(46):18322–36. 39436740 10.1021/acs.analchem.4c02645

[78] Du J, Su Y, Qian C, Yuan D, Miao K, Lee D, et al. Raman-guided subcellular pharmaco-metabolomics for metastatic melanoma cells. Nat Commun. 2020;11(1):4830. 32973134 10.1038/s41467-020-18376-x PMC7518429

[79] Kobayashi-Kirschvink KJ, CS Comiter, S Gaddam, T Joren, EI Grody, JR Ounadjela, K Zhang, B Ge, JW Kang, RJ Xavier, PTC So, T Biancalani, J Shu, A Regev. Prediction of single-cell RNA expression profiles in live cells by Raman microscopy with Raman2RNA. Nat Biotechnol. 2024;42:1726–34. https://www.nature.com/articles/s41587-023-02082-2. 10.1038/s41587-023-02082-2 PMC11233426 38200118

[80] Ryabchykov O, S Guo, T Bocklitz. Analyzing Raman spectroscopic data. Phys Sci Rev. 2019;4(2):20170043. 10.1515/psr-2017-0043.

[81] Parastar H, R Tauler. Big (Bio)chemical data mining using chemometric methods: a need for chemists. Angew Chem-Int Ed. 2022;61(44):e201801134. 10.1002/anie.201801134 29569816

[82] Chen W, Yang K, Yu Z, Shi Y, Chen CLP. A survey on imbalanced learning: latest research, applications and future directions. Artif Intell Rev. 2024;57(6):137.

