# Supplementary figures and images for: RamEx: an R package for high-throughput microbial ramanome analyses with accurate quality assessment
Source: Microbiome. 2026 Feb 10;14:89. doi: 10.1186/s40168-026-02339-3 (PMC12990539; doi:10.1186/s40168-026-02339-3)

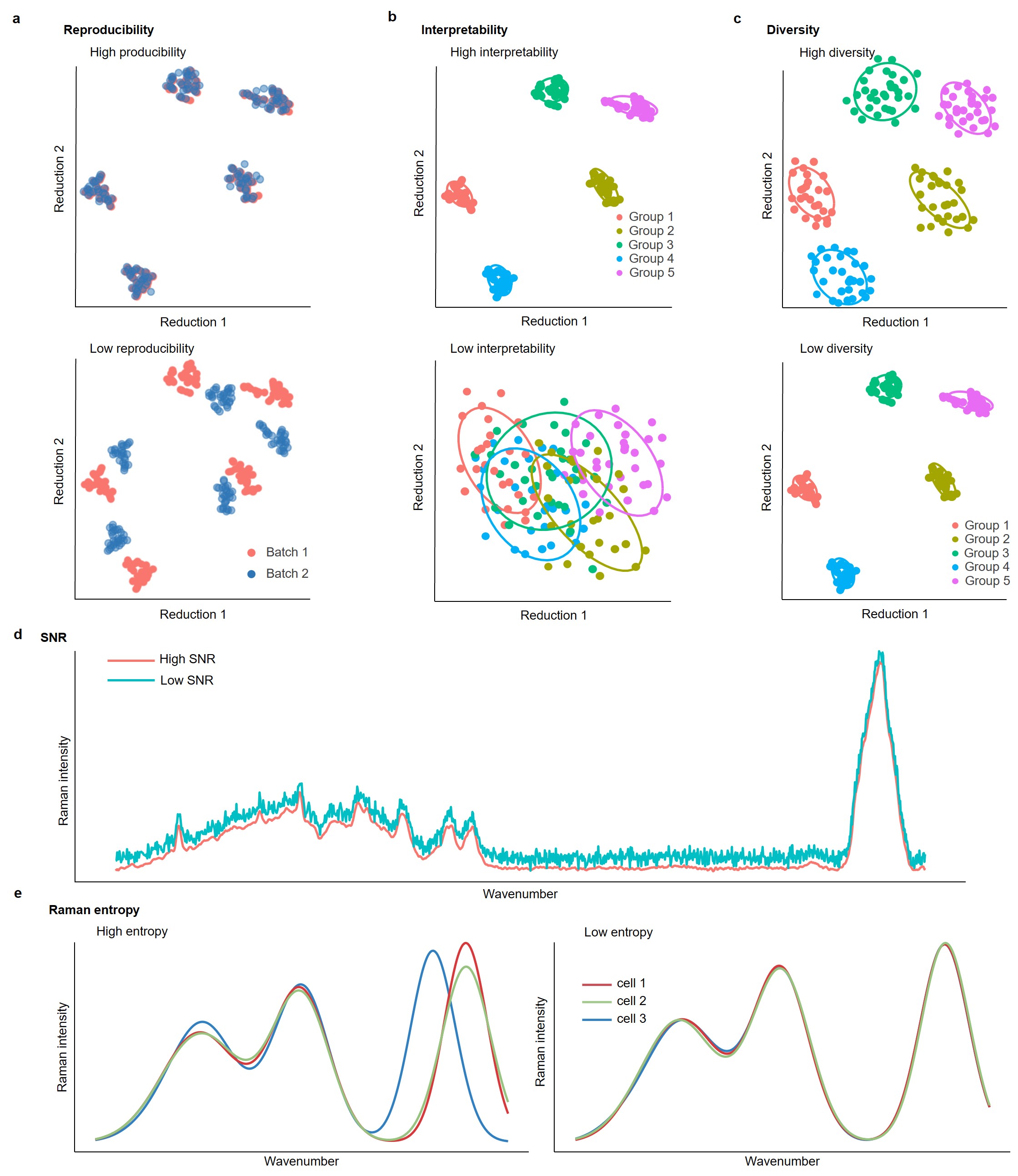

Supplement: Supplementary file 3 — Supplementary Material 2. [file 40168_2026_2339_MOESM2_ESM.zip › Figure S1.jpg]

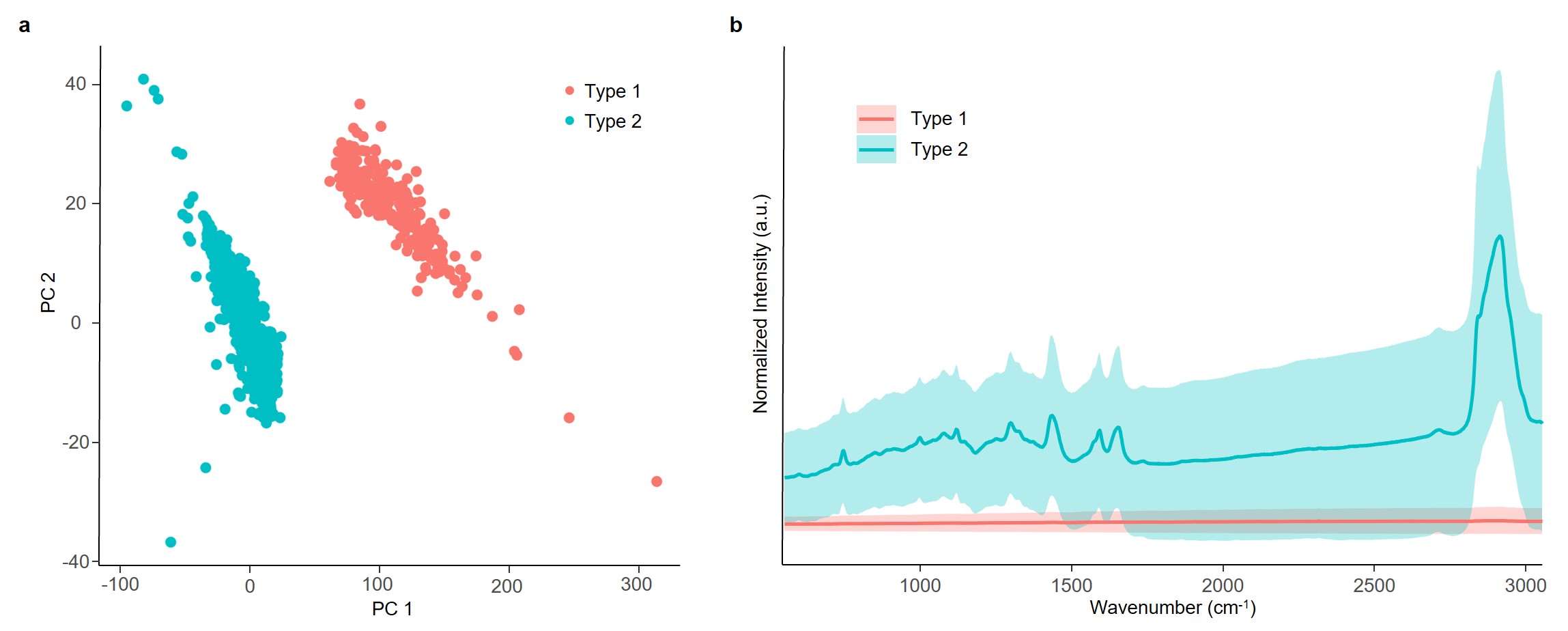

Supplement: Supplementary file 3 — Supplementary Material 2. [file 40168_2026_2339_MOESM2_ESM.zip › Figure S10.jpg]

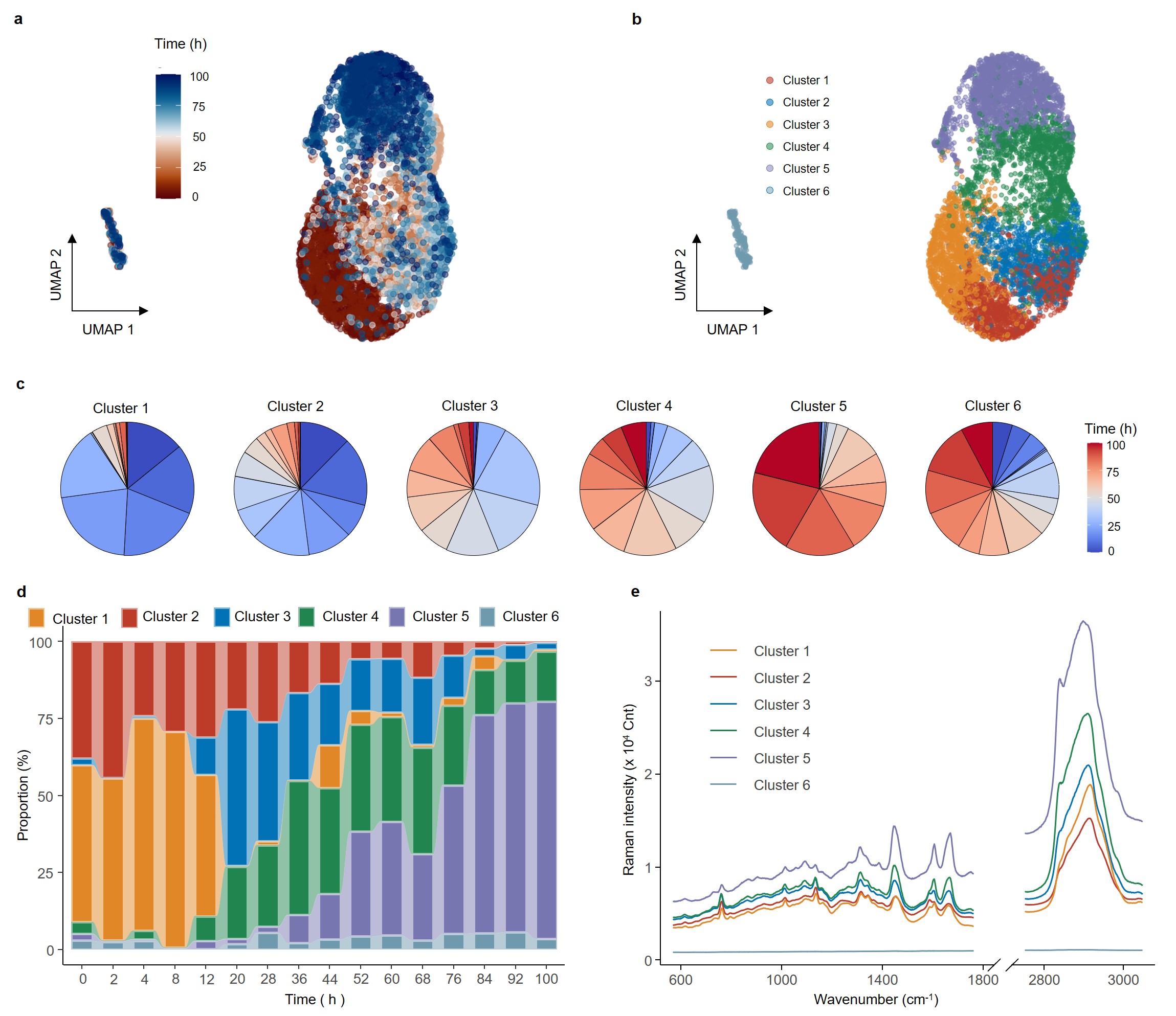

Supplement: Supplementary file 3 — Supplementary Material 2. [file 40168_2026_2339_MOESM2_ESM.zip › Figure S11.jpg]

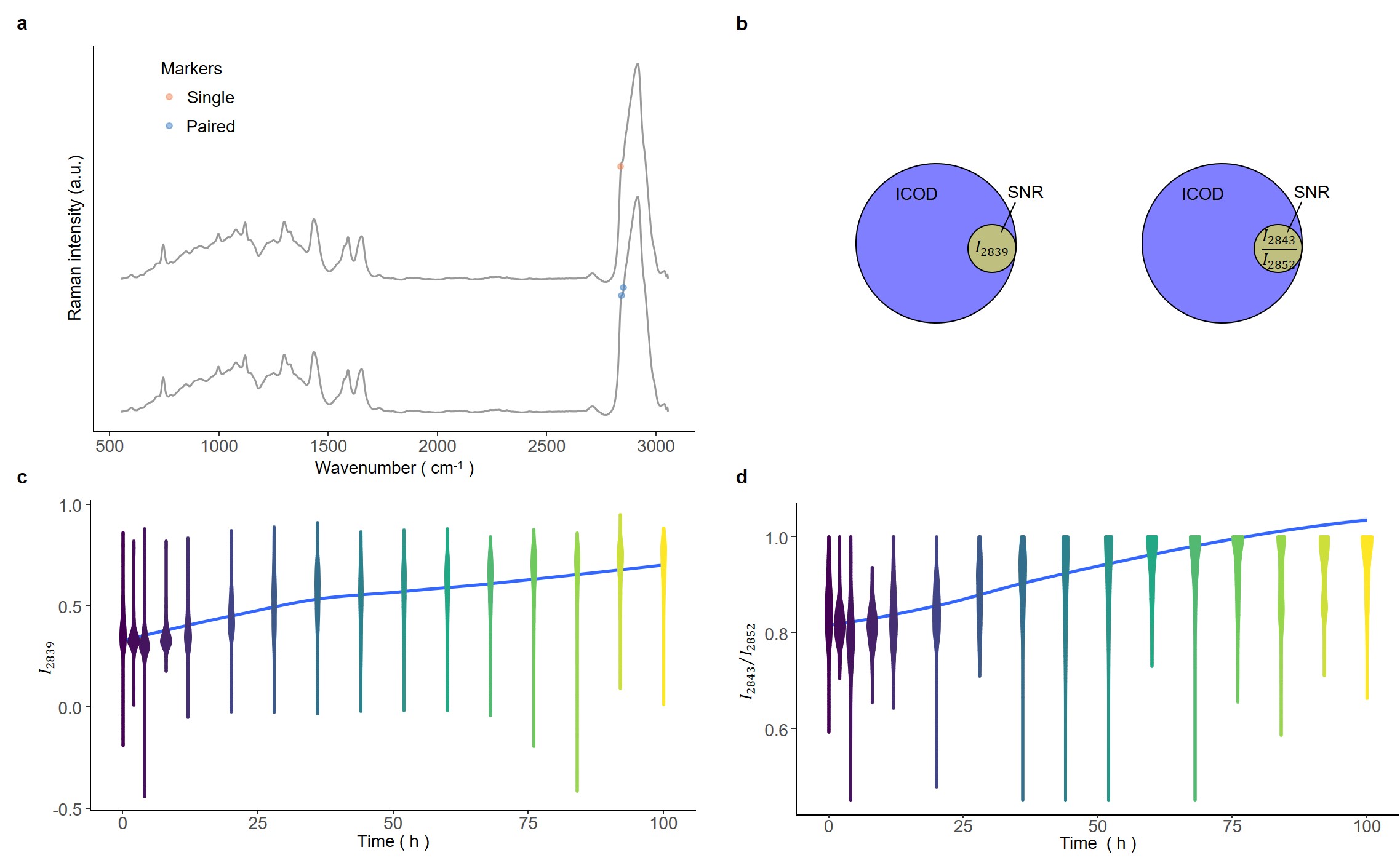

Supplement: Supplementary file 3 — Supplementary Material 2. [file 40168_2026_2339_MOESM2_ESM.zip › Figure S12.jpg]

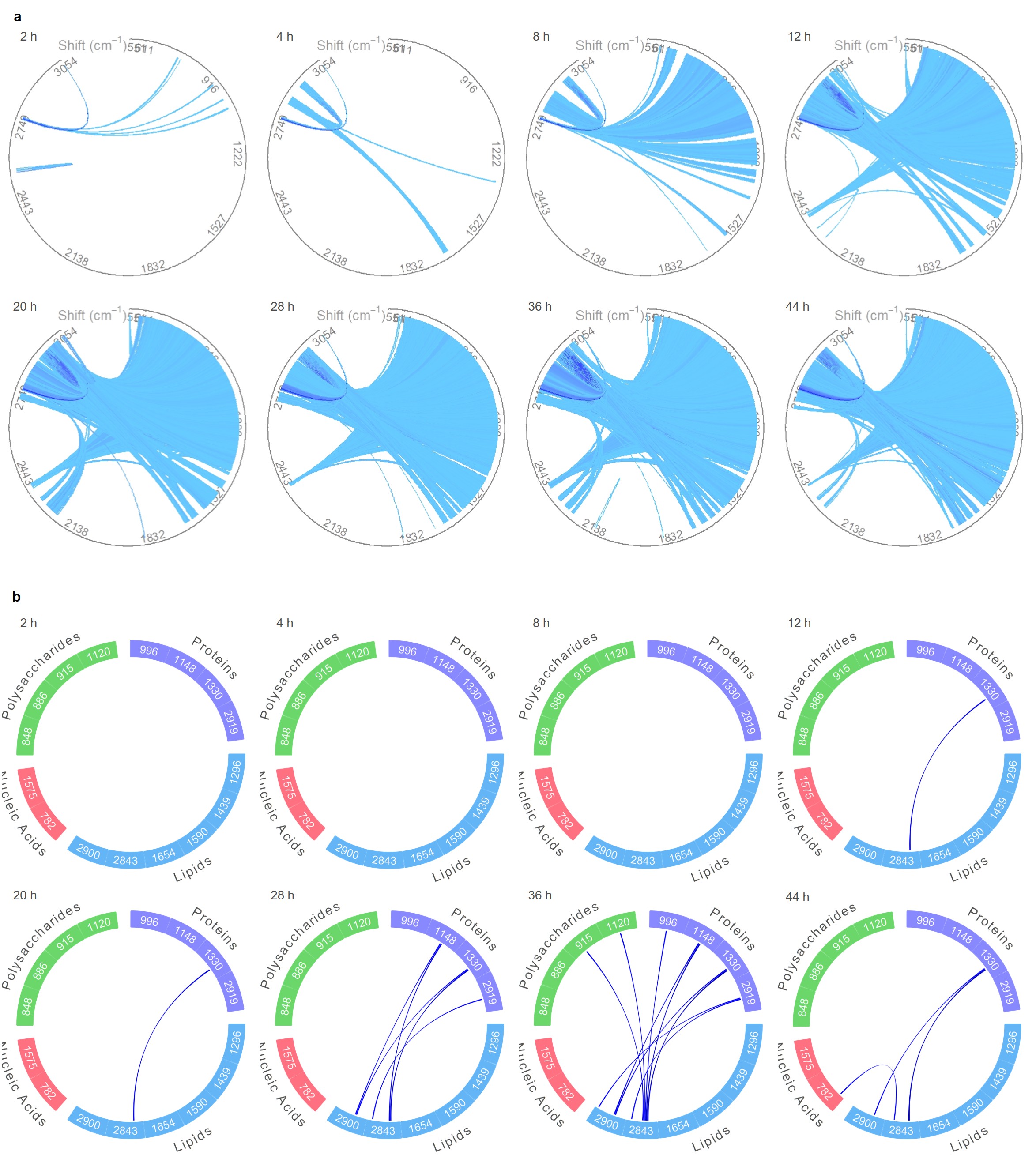

Supplement: Supplementary file 3 — Supplementary Material 2. [file 40168_2026_2339_MOESM2_ESM.zip › Figure S13.jpg]

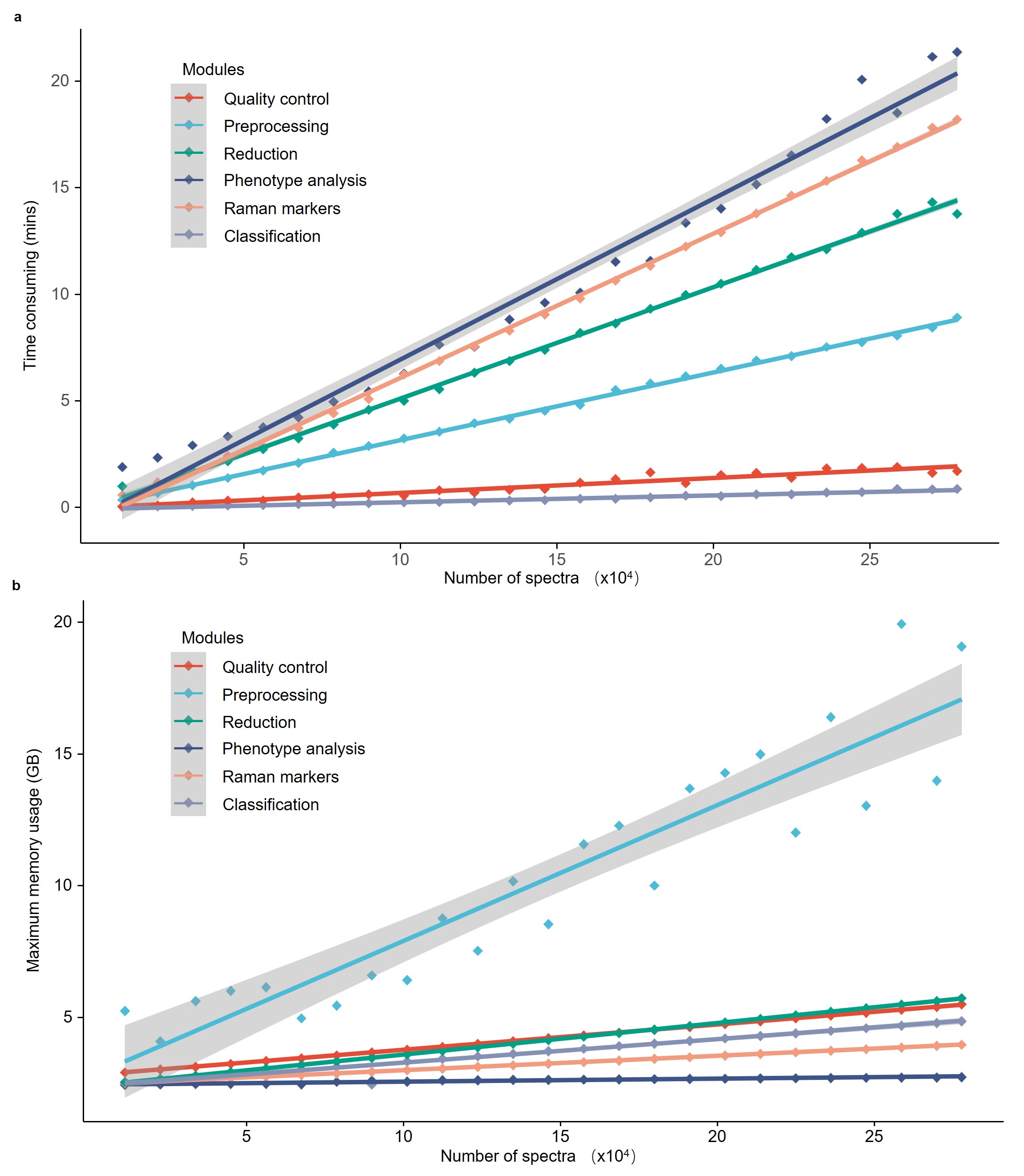

Supplement: Supplementary file 3 — Supplementary Material 2. [file 40168_2026_2339_MOESM2_ESM.zip › Figure S14.jpg]

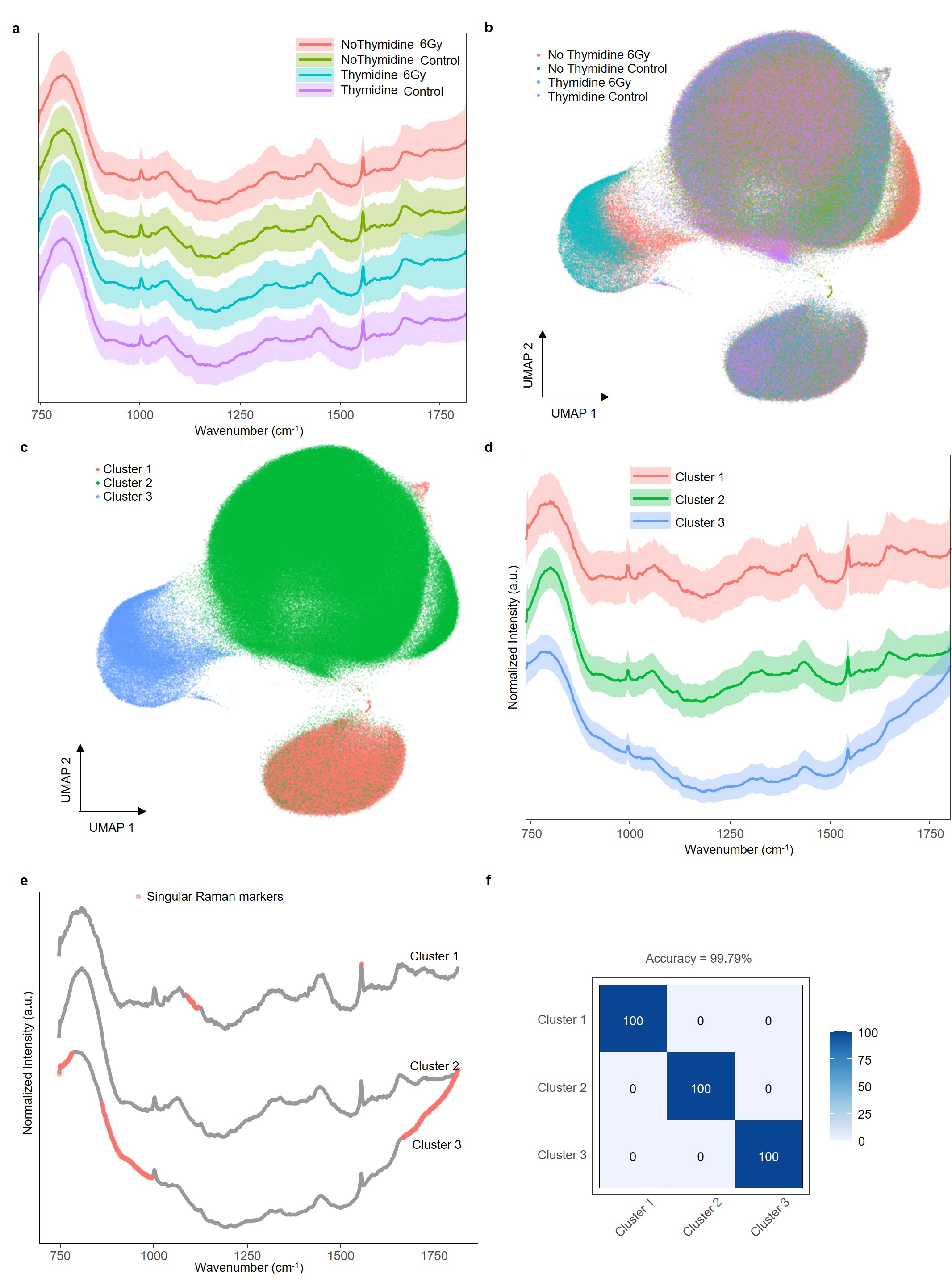

Supplement: Supplementary file 3 — Supplementary Material 2. [file 40168_2026_2339_MOESM2_ESM.zip › Figure S15.jpg]

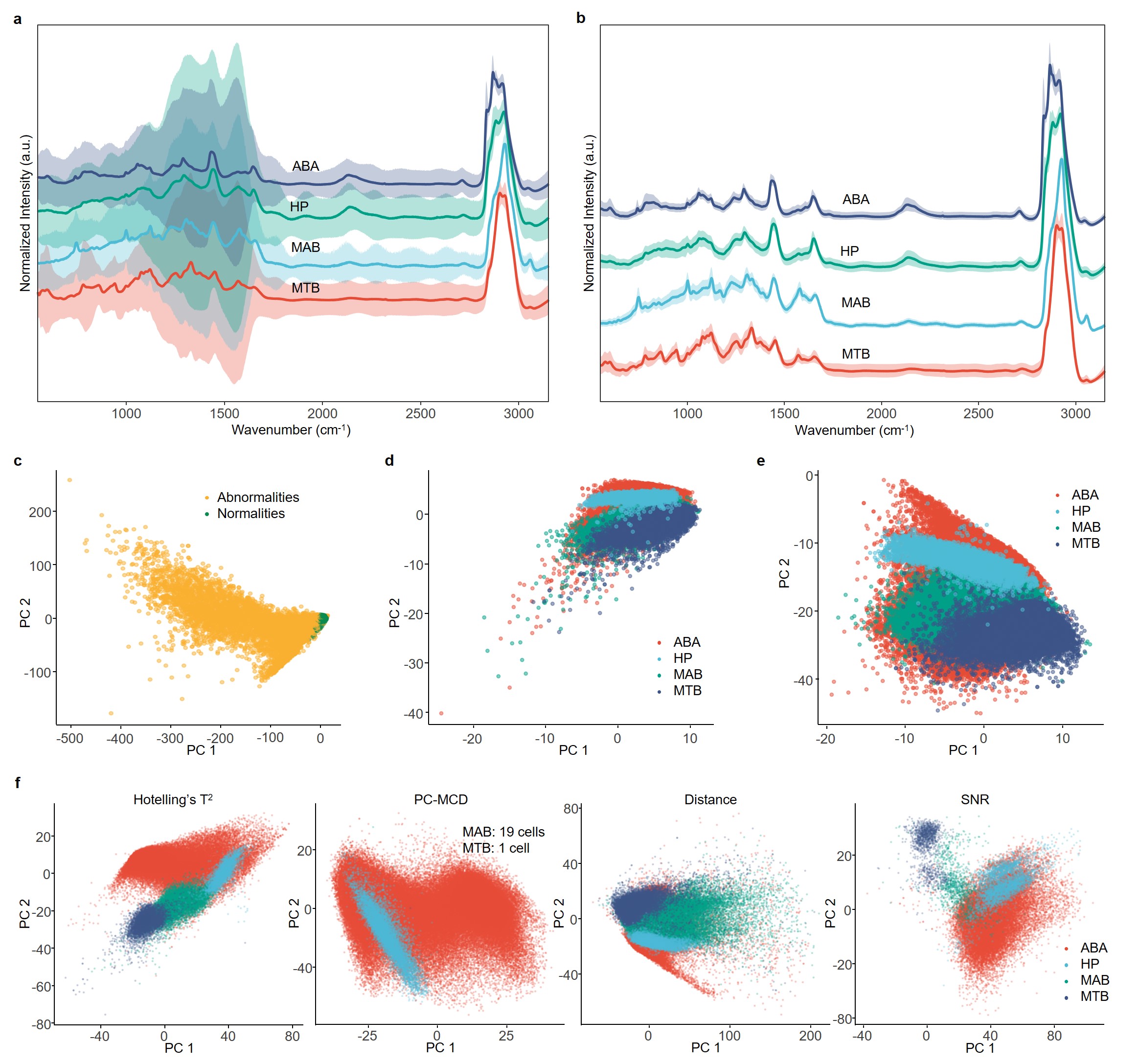

Supplement: Supplementary file 3 — Supplementary Material 2. [file 40168_2026_2339_MOESM2_ESM.zip › Figure S16.jpg]

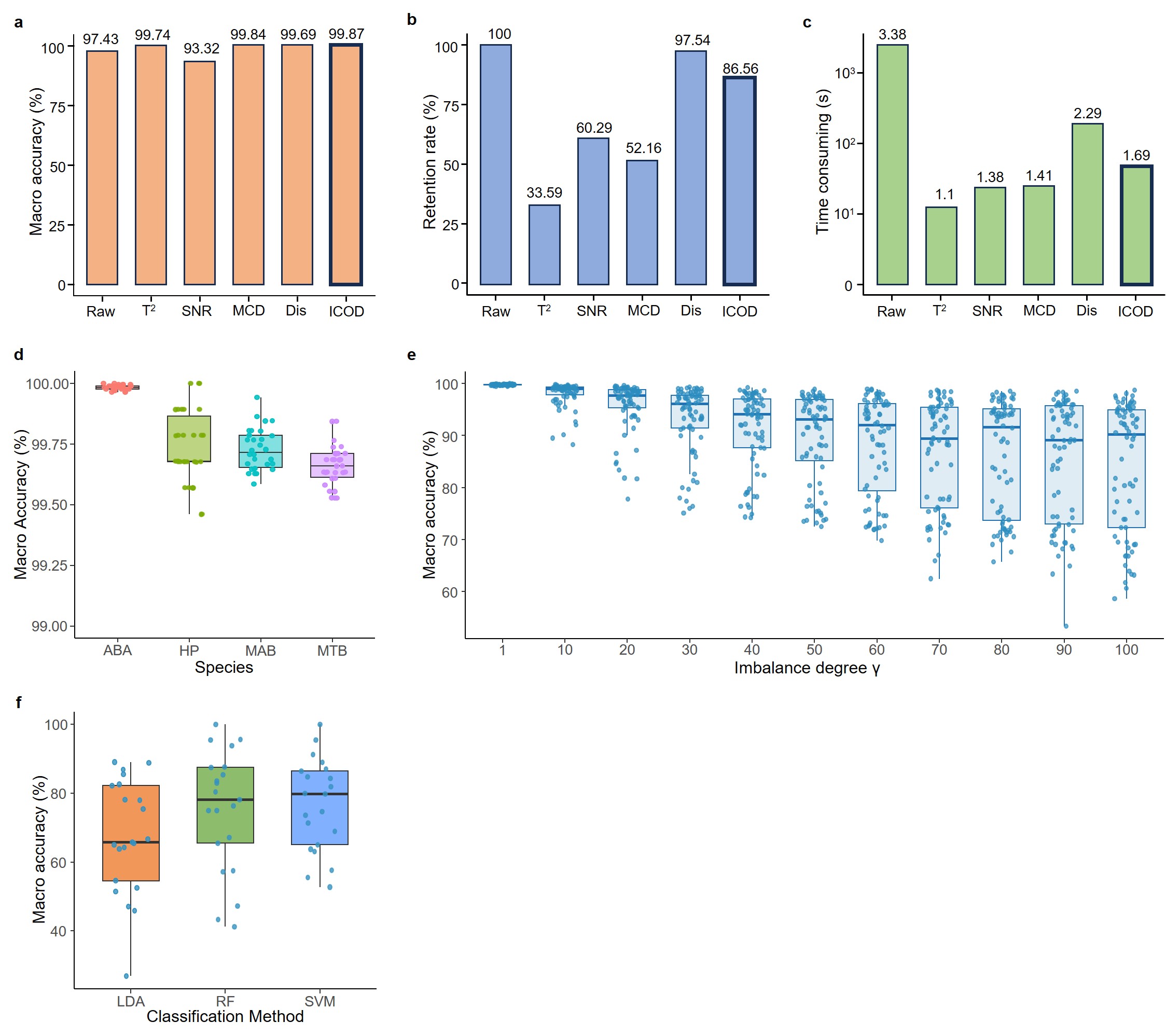

Supplement: Supplementary file 3 — Supplementary Material 2. [file 40168_2026_2339_MOESM2_ESM.zip › Figure S17.jpg]

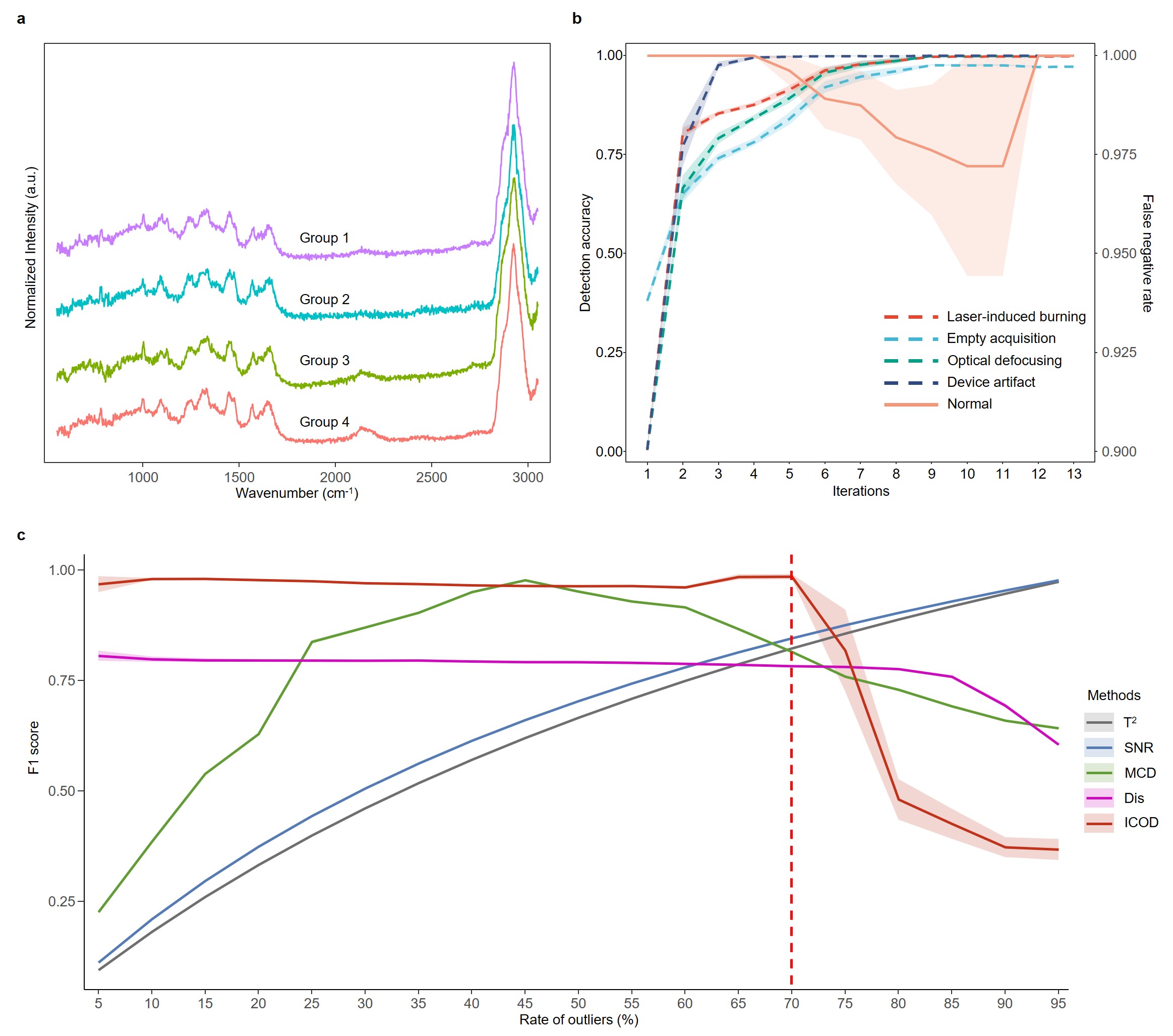

Supplement: Supplementary file 3 — Supplementary Material 2. [file 40168_2026_2339_MOESM2_ESM.zip › Figure S2.jpg]

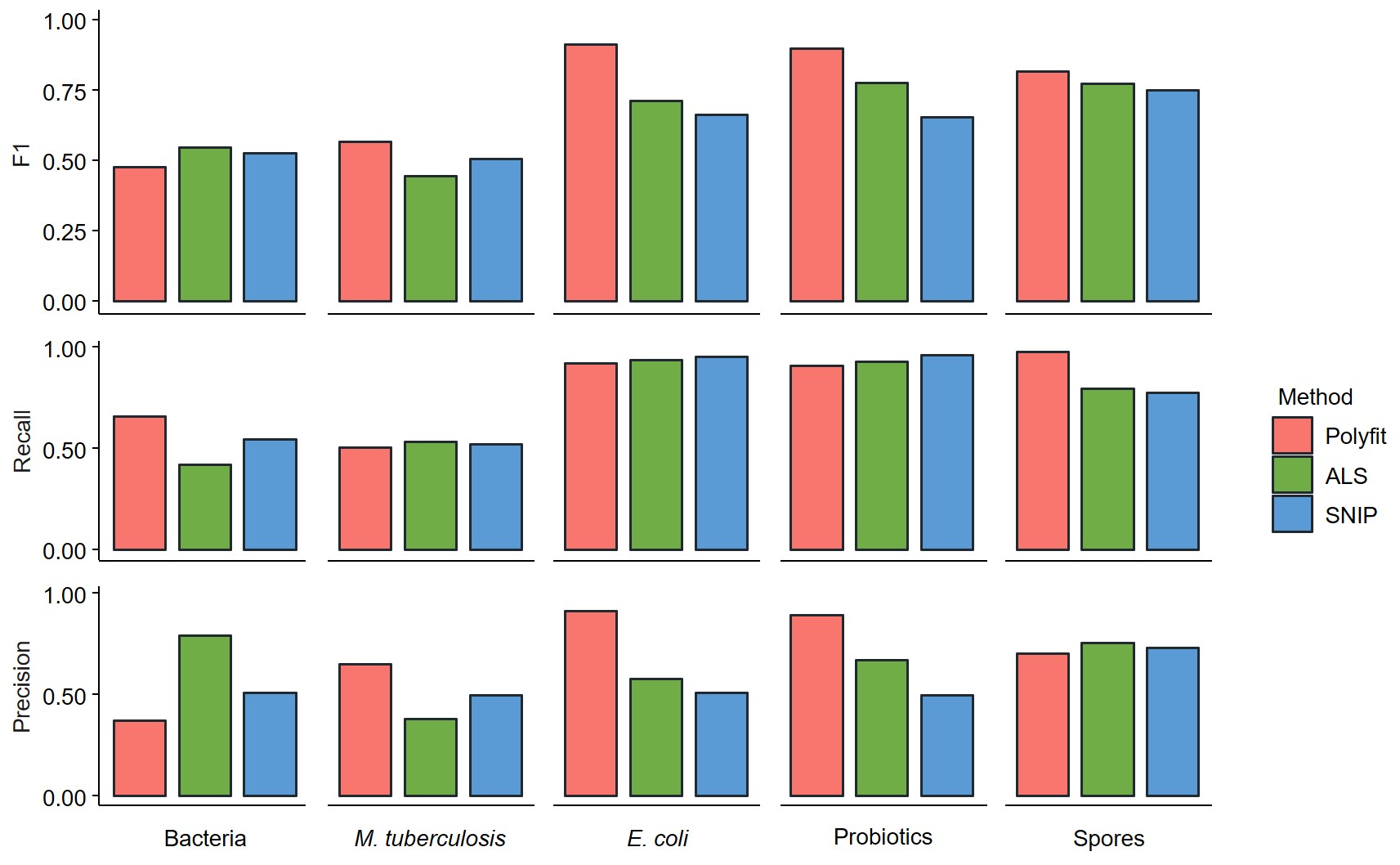

Supplement: Supplementary file 3 — Supplementary Material 2. [file 40168_2026_2339_MOESM2_ESM.zip › Figure S3.jpg]

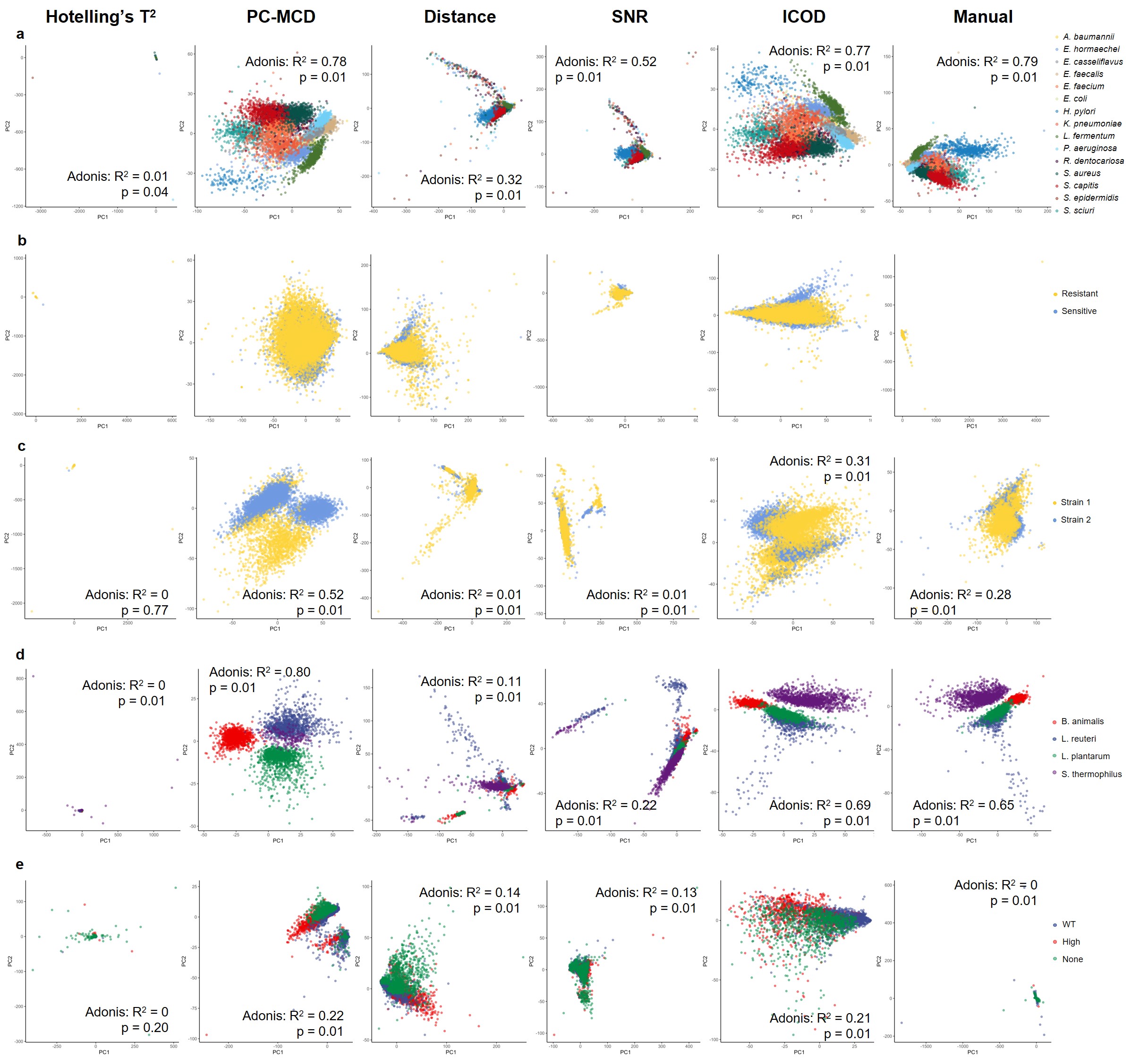

Supplement: Supplementary file 3 — Supplementary Material 2. [file 40168_2026_2339_MOESM2_ESM.zip › Figure S4.jpg]

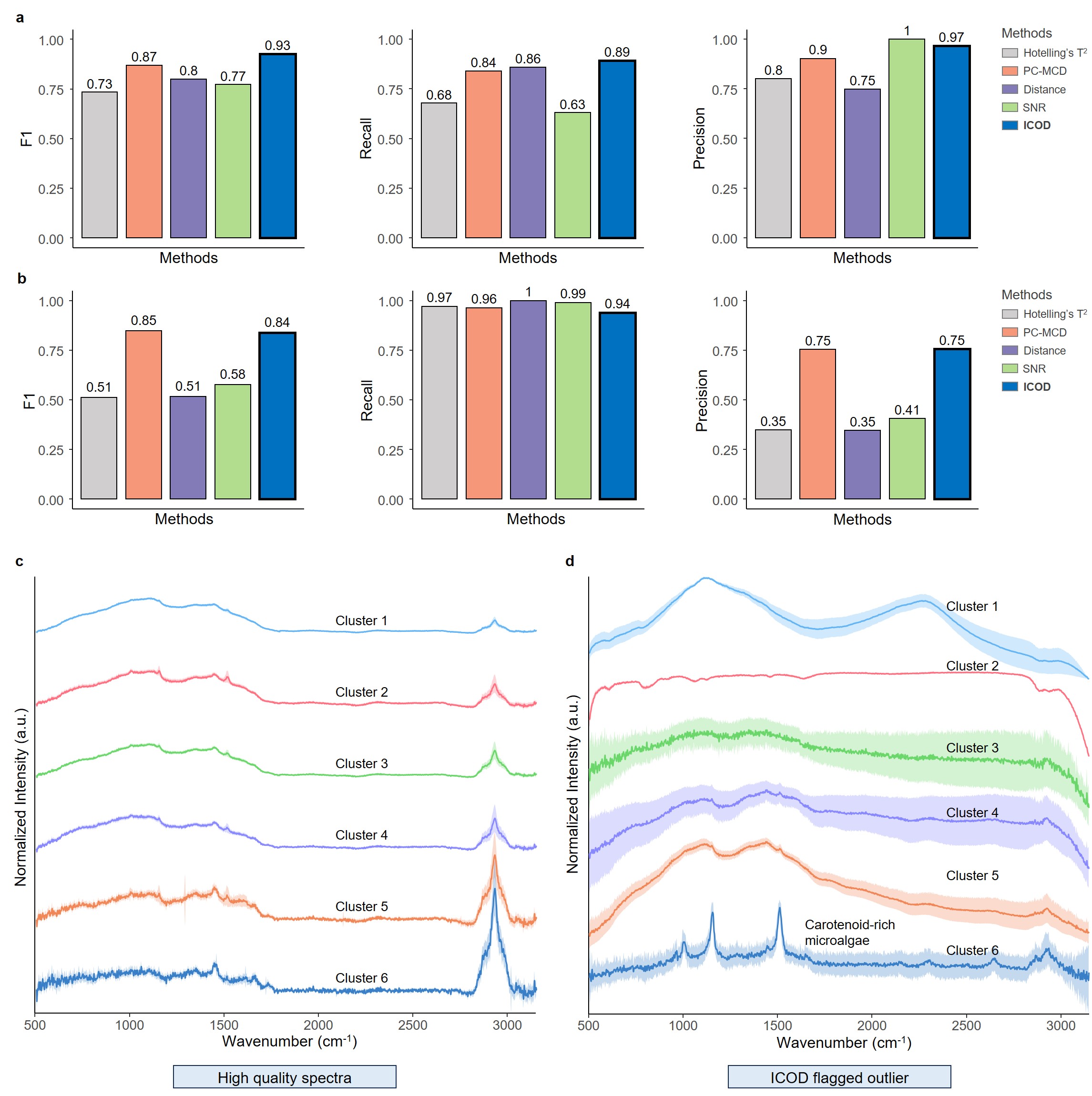

Supplement: Supplementary file 3 — Supplementary Material 2. [file 40168_2026_2339_MOESM2_ESM.zip › Figure S5.jpg]

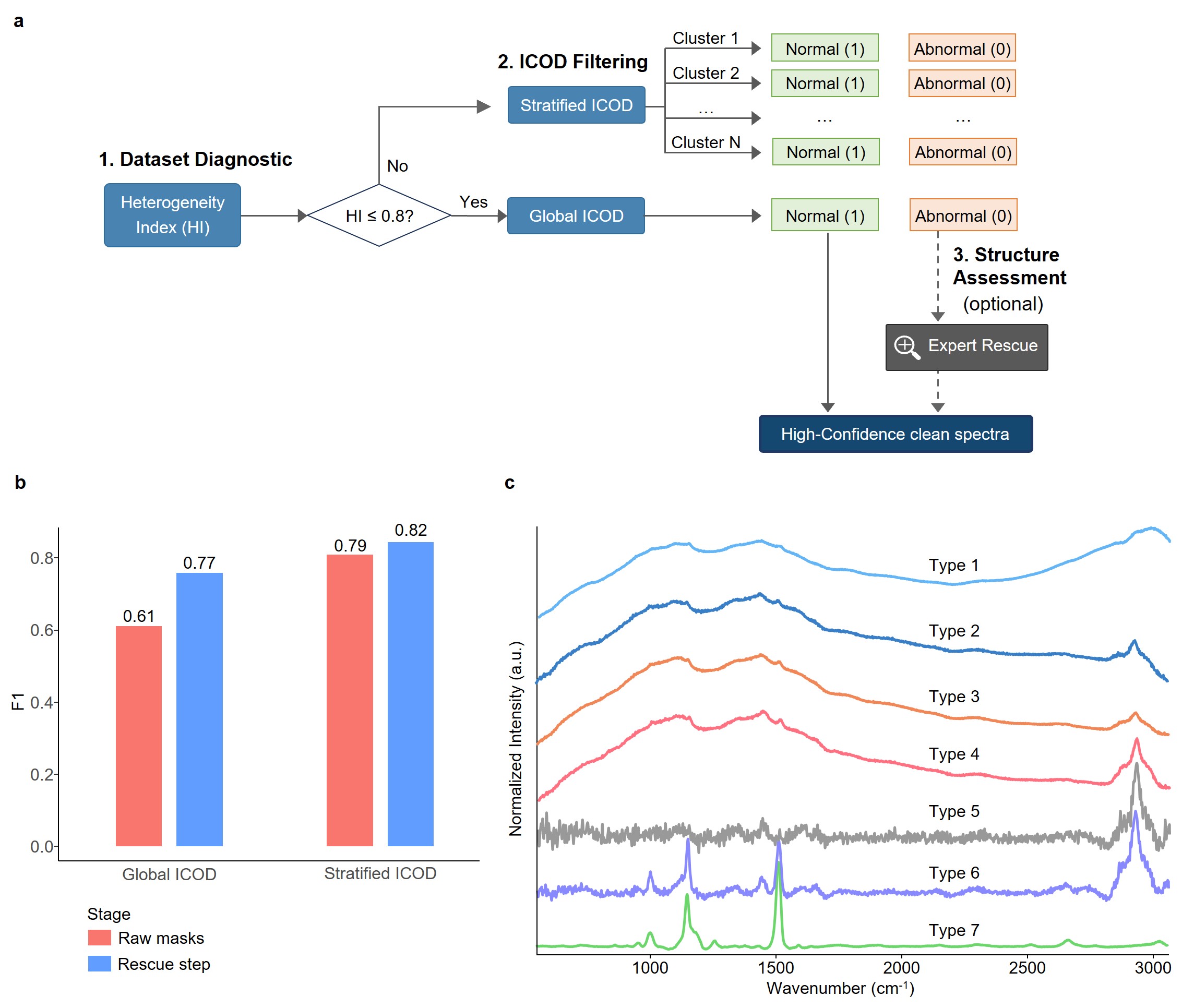

Supplement: Supplementary file 3 — Supplementary Material 2. [file 40168_2026_2339_MOESM2_ESM.zip › Figure S6.jpg]

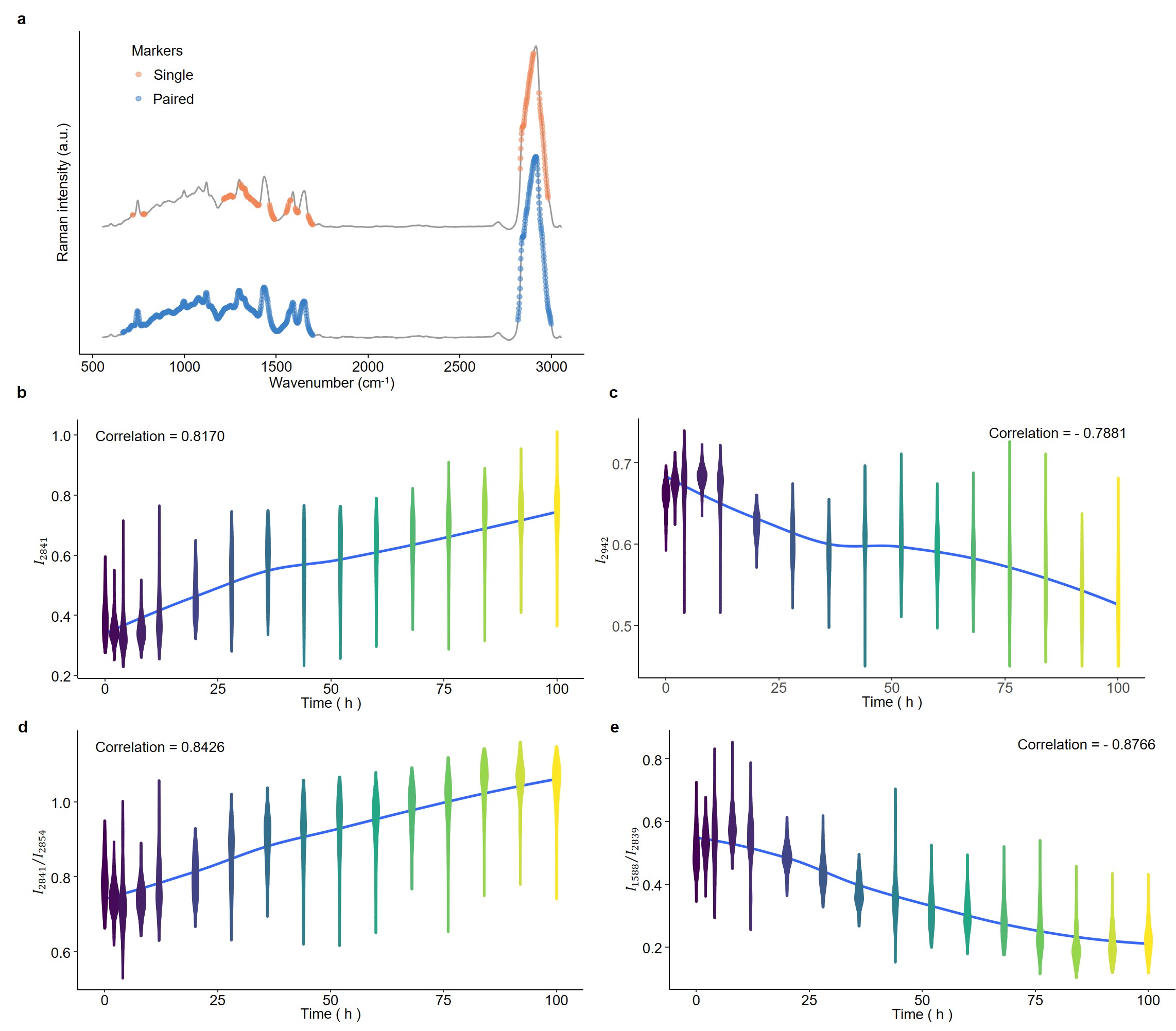

Supplement: Supplementary file 3 — Supplementary Material 2. [file 40168_2026_2339_MOESM2_ESM.zip › Figure S7.jpg]

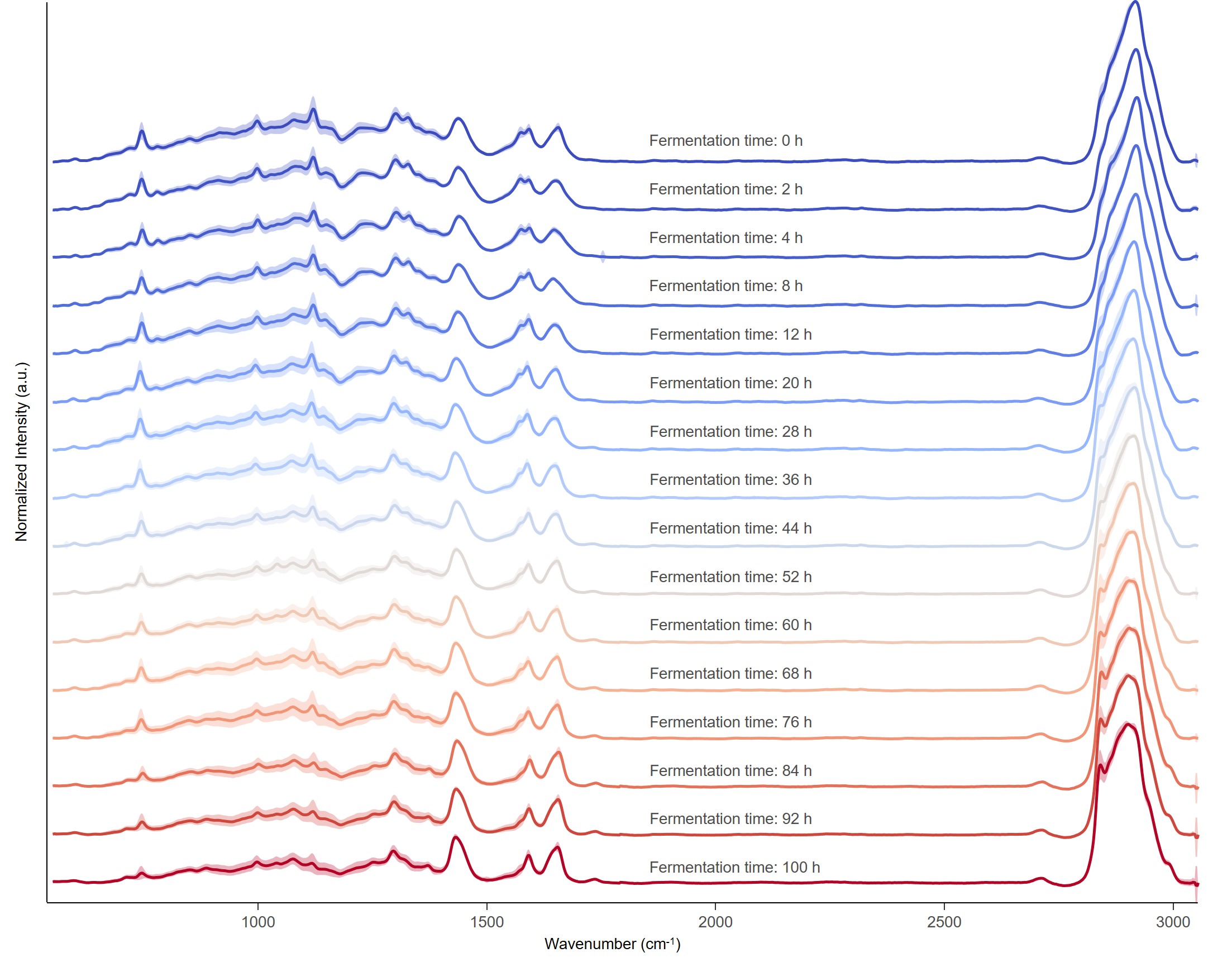

Supplement: Supplementary file 3 — Supplementary Material 2. [file 40168_2026_2339_MOESM2_ESM.zip › Figure S8.jpg]

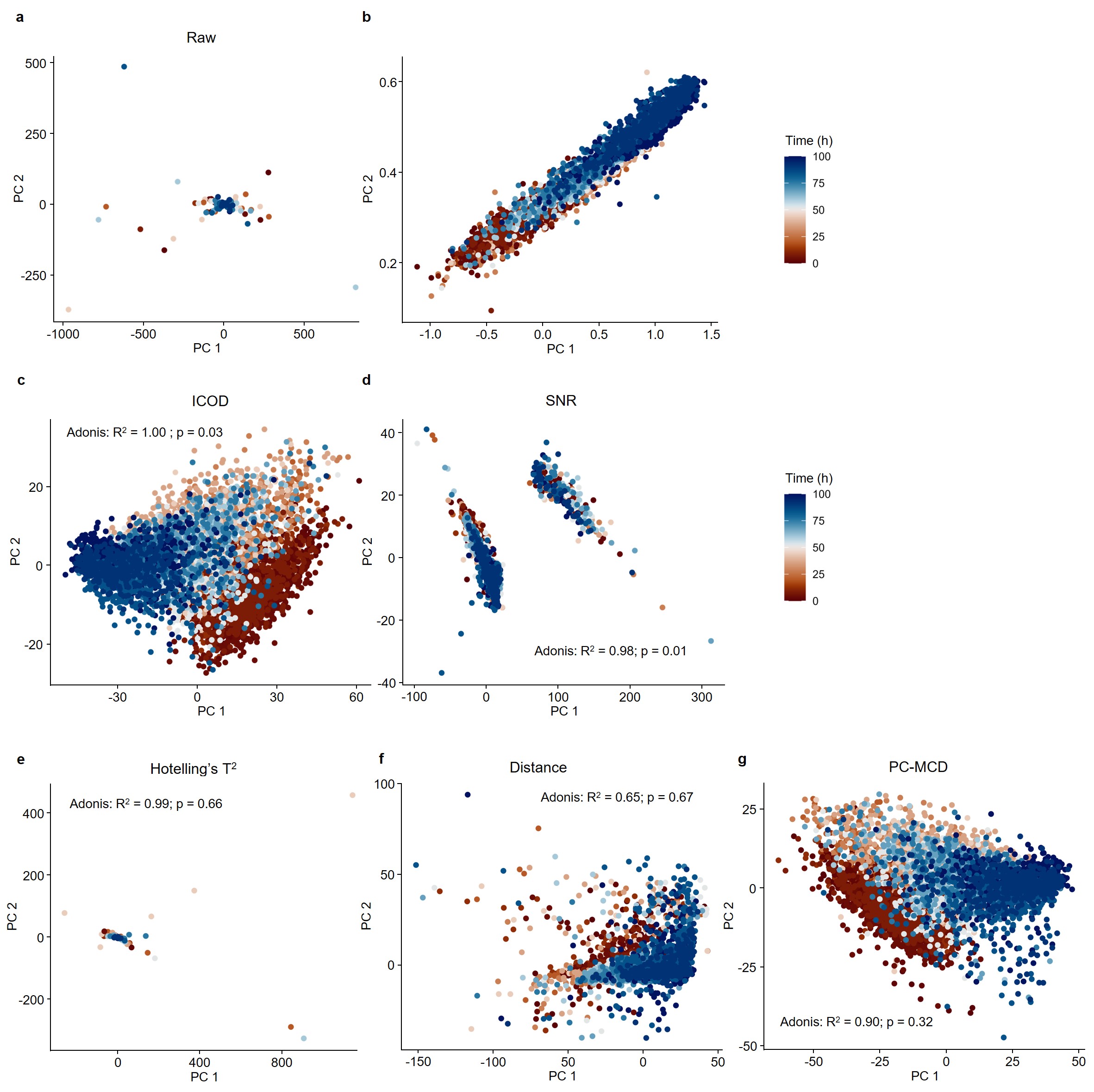

Supplement: Supplementary file 3 — Supplementary Material 2. [file 40168_2026_2339_MOESM2_ESM.zip › Figure S9.jpg]

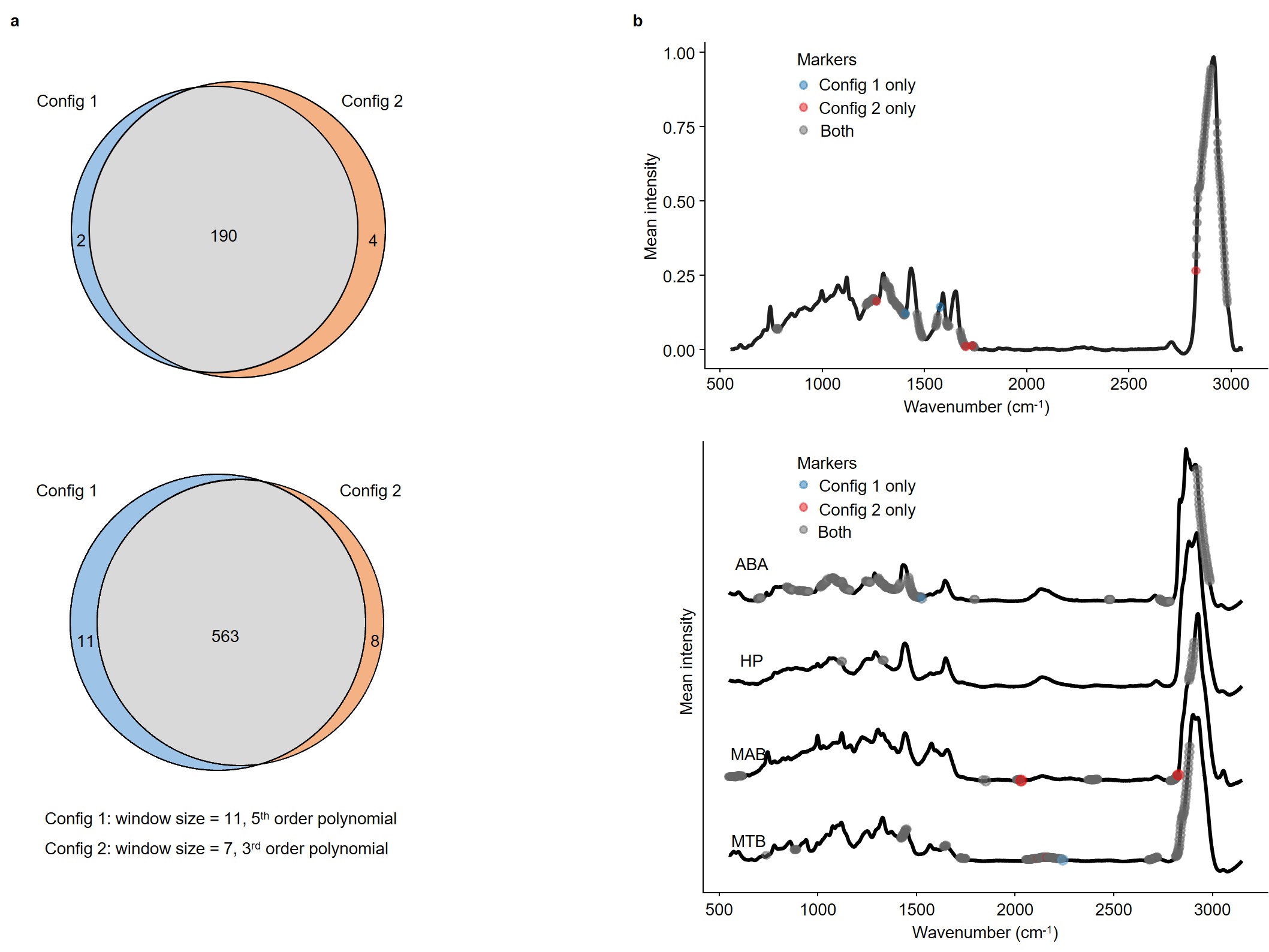

Supplement: Supplementary file 3 — Supplementary Material 2. [file 40168_2026_2339_MOESM2_ESM.zip › Figure_S18.jpg]

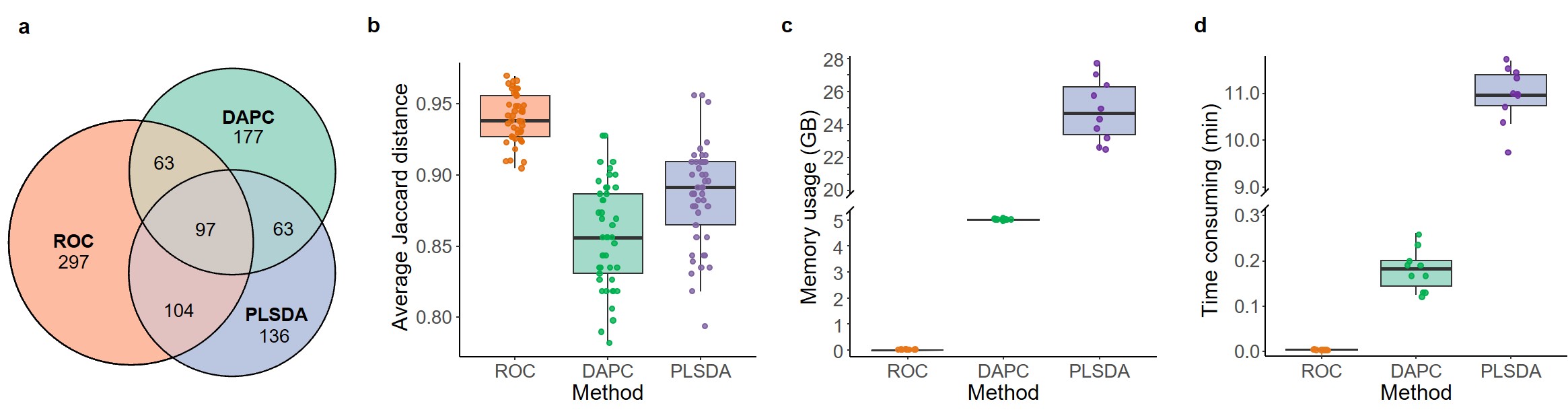

Supplement: Supplementary file 3 — Supplementary Material 2. [file 40168_2026_2339_MOESM2_ESM.zip › Figure_S19.jpg]
